# Ion transport mediated pH-responsive MRI contrast in dual wrapped MSNs

**DOI:** 10.1039/d6sc05306h

**Published:** 2026-07-20

**Authors:** James P. Smith, Anna M. Duncan, Connor M. Ellis, Aidan Kerckhoffs, Matthew J. Langton, Jason J. Davis

**Affiliations:** a Department of Chemistry, University of Oxford South Parks Road Oxford OX1 3QZ UK Jason.davis@chem.ox.ac.uk matthew.langton@chem.ox.ac.uk +44 (0)1865 275 914

## Abstract

Functionalised polymers act as environmentally-responsive coatings, capable of sterically gating water exchange between bulk and Gd(iii) centres within mesoporous silica nanoparticles (Gd-MSNs). Using poly(2-(dimethylamino)ethyl methacrylate) (pDMAEMA, p*K*_a_ = 6.4) this water barrier is pH actuated (MRI ‘on’ at pH < p*K*_a_, MRI ‘off’ at pH > p*K*_a_). Such pH-responsive polymer coated Gd-MSNs can subsequently be enveloped within vesicular structures, further limiting water access, providing an improved MRI ‘off’ state for the so-generated dual-wrapped structure (*r*_1_ = 2.4 ± 0.2 mM^−1^ s^−1^, pH 8.0, 1.41 T, 298 K) compared with the polymer alone. When ion carriers (here a chloride-transporting tripodal thiourea alongside a protonophore, carbonyl cyanide *m*-chlorophenyl hydrazone) are integrated, it is possible to couple this pH gating to specific symport ion transport such that, ultimately, ionic rcognition triggers a ∼500% magnitude enhancement in relaxivity (*r*_1_ = 14.5 ± 0.8 mM^−1^ s^−1^ at pH 5.0).

## Abstract 2

A pH-responsive polymer and lipid bilayer cooperatively gate water exchange in Gd-doped mesoporous silica nanoparticles, generating a highly suppressed MRI ‘off’ state. Ion carrier-mediated H^+^/Cl^−^ symport re-establishes water access at low pH, enhancing relaxivity by ∼500%.

## Graphical abstract

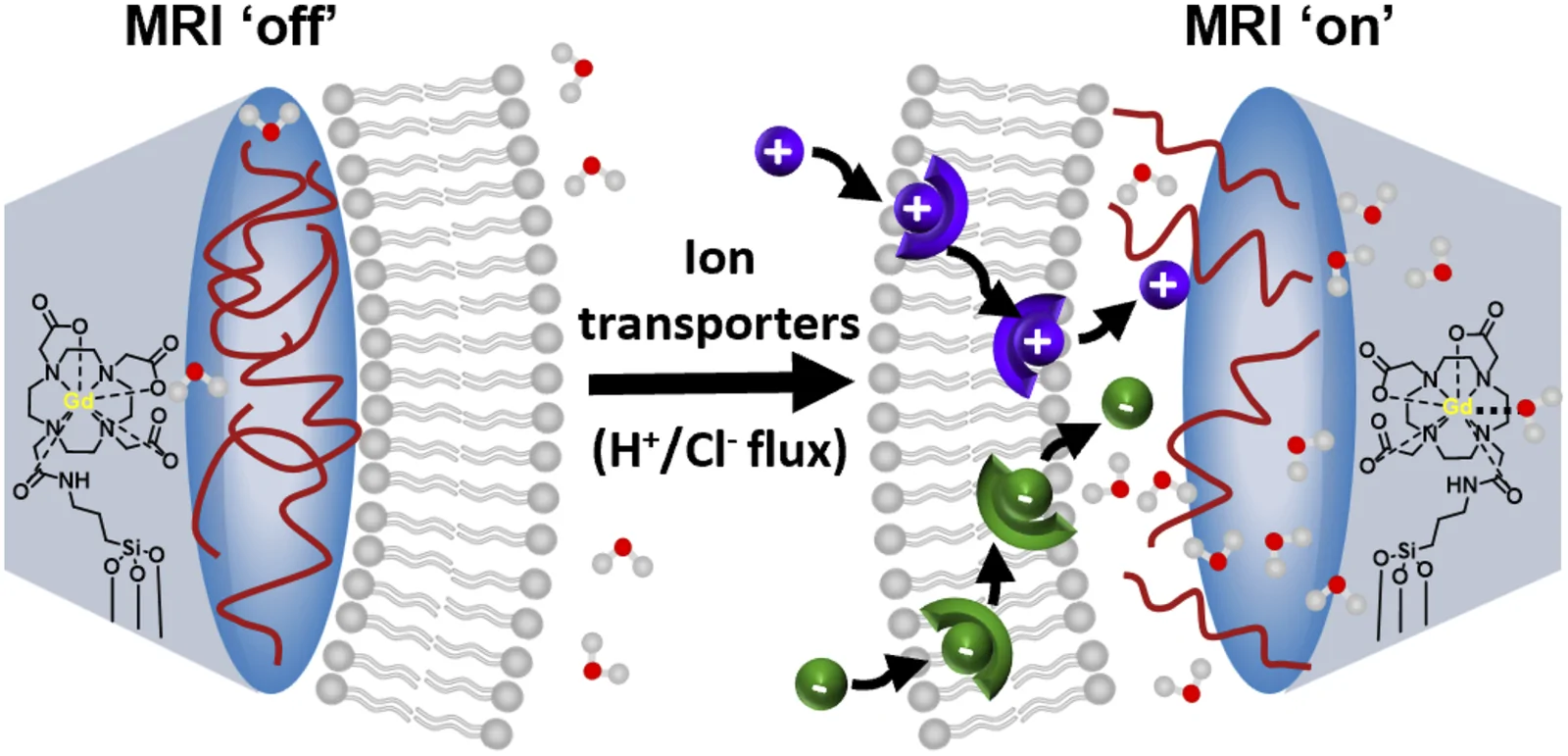

## Introduction

Nanoparticle-based MRI contrast agents (CAs), including superparamagnetic iron oxide nanoparticles (SPIONs), gold nanoparticles (AuNPs), and Mn-/Ln-doped nanoparticles achieve enhanced relaxivity values compared with molecular chelates, owing to favourable rotational characteristics.^1–4^ Among these, lanthanide-doped mesoporous silica nanoparticles (Ln-MSNs) are particularly attractive due to accessible surface chemistry, enabling facile and efficient paramagnetic loading that is both well-defined and locally tuneable.^5,6^ The specific tethering of paramagnetic chelates within the mesopores can be followed by a molecular gating of pore confined water through post-synthetic modification with environmentally-responsive motifs (*e.g.*, polymers).^7–9^

Within the Solomon-Bloembergen-Morgan (SBM) framework, the inner sphere (IS) contribution to relaxivity is well established to be significant in optimising resolved values, governed by *q* (hydration number), water residence time (*τ*_M_), and rotational correlation time (*τ*_R_). The introduction of organic motifs, *e.g.*, polymeric shells or lipid bilayers, can modulate *r*_1_ through significant changes in the local water pool accessible to integrated paramagnetic chelates.^7^

Valuable targets for a responsive contrast modality include an ability to define disease pathology typified by cancerous tissue: that is an abnormally acidic extracellular pH range of 6.3–7.0 (normal tissue ∼7.4), elevated temperature, hypoxia, and dysregulated specific ion presence.^10,11^ These pathological characteristics largely arise from a high glycolytic rate and dysregulated gene expression.^12^ MRI CAs have, accordingly, been engineered to respond selectively to thermal, pH, and hypoxic triggers, generally by fragmentation approaches, gating of water access, or a triggered change in IS hydration number (*q*).^13–15^

Paramagnetic chelates bound to rigid scaffolds are known to promote water relaxation in a more efficient manner than traditional GBCAs.^16^ Molecular coupling to the rigid particle scaffold restricts chelate rotation, allowing for more effective coupling to water (aligning with the Larmor precession), enhancing derived relaxivity values.^17^ In this work, we bind chelates within silica mesopores that are exposed to a highly limited volume of water, facilitating control over the exchange of this limited pool with bulk through separate pH- and ion-responsive barriers. In the design of specific stimuli-responsive nanoparticle CAs, pH-sensitive polymer coatings with low associated cytotoxicity have proved to be effective, and customisable, in responding to both acidic and basic microenvironments.^18,19^ The polymer coat modification of a paramagnetic core enables an initial MRI ‘off’ state by gating water accessibility. In prior work, we have shown, for example, that a poly(methacrylic acid) coating (pMAA, p*K*_a_ = 5.2) grafted from Gd-MSNs can generate a significant reversible MRI contrast switch as the local environment is switched from acidic to basic, driving an increase in *r*_1_ of 182% at 1.5 T.^8^ In contrast, pDMAEMA represents a widely applied polybasic coating whose p*K*_a_ (6.4) falls within a physiologically relevant range for investigating conditions that include cancer (extracellular pH = 6.3–7.0) or atherosclerosis (pH < 6.0).^12,20–22^ A post-synthetic polymer modification of MSNs has been shown in many instances to improve circulation times, targeting, and to have minimal *in vivo* cytotoxicity.^1,23,24^

An environmentally-responsive capacity has also been demonstrated by wrapping paramagnetic chelates, or paramagnetically-doped particles, in biocompatible phosphatidylcholine (POPC) lipid bilayers.^25–27^ In such configurations, responsivity is achieved because of the low native levels of contrast supported by the very small water pool within the vesicular lumen, and low water flux across the bilayer.^28,29^ We have shown that subsequently activating an ionophore-mediated transmembrane ionic flux, with its associated hydration,^28^ supports an ionically-selective concomitant switch ‘on’ in MRI contrast.^30–33^

**Fig. 1:**
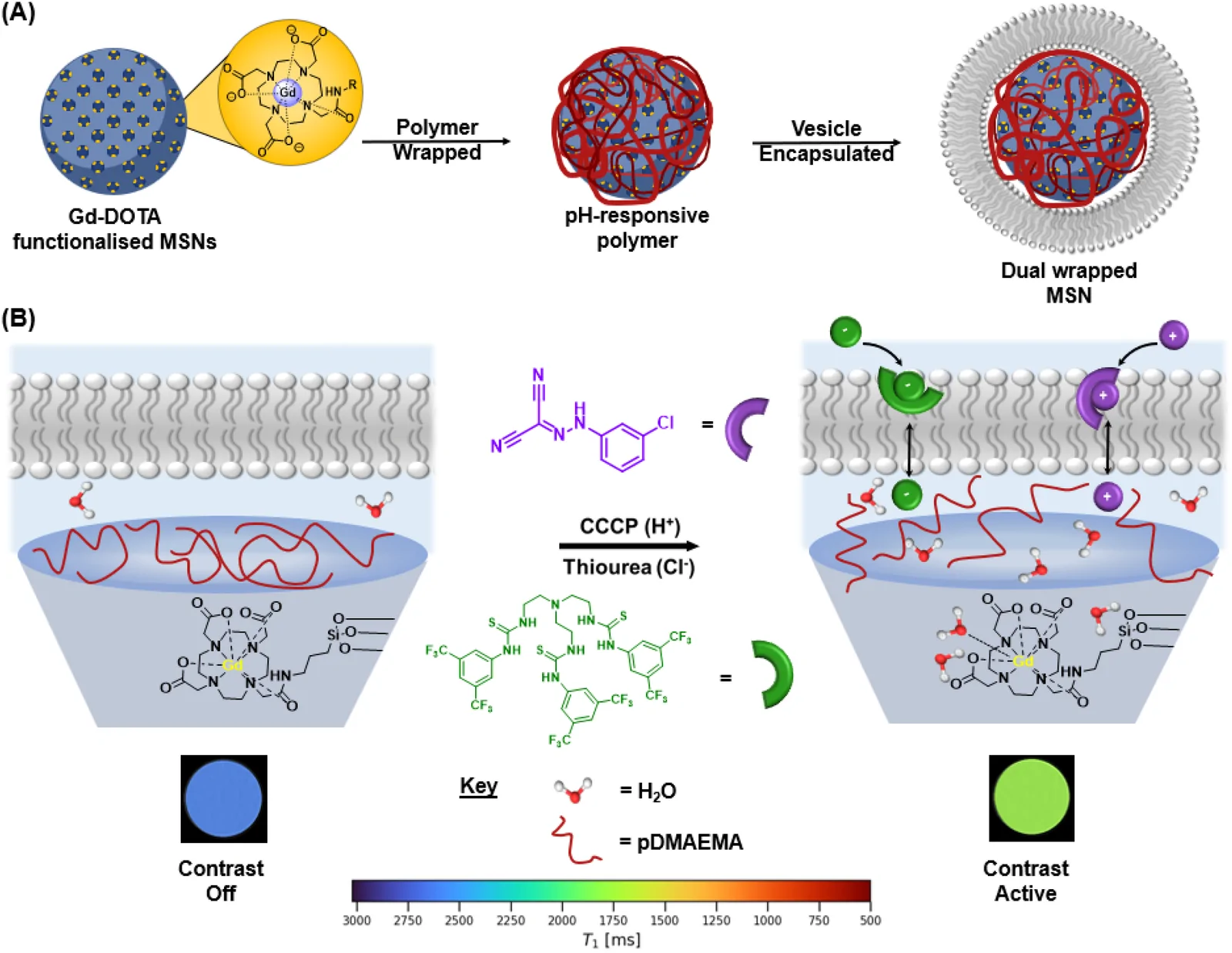
(A) A Gd-MSN is first electrostatically wrapped with a pH-responsive polymer and collected, yielding a pH sensitive nanoparticulate CA. The p-Gd-MSN is then encapsulated within a cholesterol doped POPC vesicle. (B) The LB-p-Gd-MSN is initially MRI ‘off’ as the lipid bilayer and deprotonated polymer coating block proton flux and water exchange, respectively. The co-integration of a protonophore, CCCP, and anionophore, tripodal thiourea, enables the pH-gradient driven symport of H^+^/Cl^−^, causing the protonation of the pDMAEMA coating and the subsequent desorption from the particle surface, allowing for increased water exchange into the pores, aided by the anion transport-driven water flux across the lipid bilayer. MRI contrast is only fully activated upon integration of both transporters and exposure to an acidic environment of pH < p*K*_a_.

To date, there are no reports detailing the cooperative integration of both polymeric and liposomal barriers within a single MRI contrast agent architecture. This is of significant interest due to the enhanced suppression of baseline relaxivity that can be achieved, alongside the opportunity for tailored responsiveness to transmembrane ion and proton flux through modification of the polymer. In this work we demonstrate that pH-responsive polymer wrapped Gd-MSNs can be incorporated within lipid bilayer vesicles to generate a highly suppressed MRI ‘off’ state in the form of a double-wrapped MSN (*i.e.*, polymer and lipid bilayer, Fig. 1). The subsequent dual integration of a protonophore and an anionophore facilitates coupled H^+^/Cl^−^ transfer across the bilayer membrane upon exposure to a low pH microenvironment. As the proton gradient is equilibrated across the bilayer, the pDMAEMA coating becomes protonated and swells (as the internal pH traverses the p*K*_a_), enhancing water accessibility to the pore-confined paramagnetic centres. *In vivo*, high extracellular chloride concentrations (∼110–120 mM) would enable a selective ‘switch on’ of contrast only in microenvironments with a sufficiently acidic pH (∼6.4), such as malignant tumour sites.^34^ This gating of molecular water by both bilayer and polymer coatings around Gd-MSNs is mechanistically novel, supporting a direct coupling of ionic and pH responsivities, demonstrating a quasi-reversible very high magnitude relaxometric response. The combination of specific background anion presence alongside abnormal pH levels inherently provides a route to enhanced specificity in the detection of targeted disease states (as well as very high magnitude switches), which can be expanded to include atherosclerosis, chronic inflammation, and ischaemia.^35–37^

## Results and discussion

Gd(iii)-loaded MSNs were first synthesised by a modified Stöber method as described in prior work (see SI 1–4 for full experimental details and characterisation).^38^ The particles were aminated at 0.3 mol% with respect to the total silane molarity, providing modifiable anchor sites to covalently tether Gd-DOTA (gadolinium(iii) 1,4,7,10-tetraazacyclododecane-1,4,7,10-tetraacetic acid).^5^ The particles displayed a high relaxivity of ∼27 mM^−1^ s^−1^ (in line with prior work),^5^ and a dynamic light scattering (DLS) resolved number mean hydrodynamic size of 98 ± 1 nm, with excellent particle uniformity (polydispersity, PDI, < 0.05). Particle pore size was determined to be 3.2 ± 0.3 nm (by BJH analysis, SI 2). An arsenazo III assay was initially utilised to verify that there was a minimal presence (0.005%) of unchelated Gd(iii) adsorbed at the MSN interface, (SI 3, 4) generating, at most, a contribution of 0.5% to the total relaxivity. It is known that Gd-MSNs without functionalised coatings are not pH-responsive (SI 5).^8^ Hence, post-synthetic modification of the MSN with pDMAEMA (denoted p-Gd-MSN) afforded the pH-sensitive and MRI-active organic–inorganic architecture.

Two synthetic approaches for the polymer wrapping of MSNs were investigated; both an electrostatic association of pDMAEMA to the paramagnetically-doped MSN surface, and a “grafting-from” the MSN interface by a surface-initiated reversible addition–fragmentation chain transfer (SI-RAFT) polymerisation (see SI 6–17 for full analyses).^39^ The integrity and thickness of the polymer coatings, in all cases, were characterised by UV-vis, ATR-IR, TGA, DLS and TEM (see SI). MSNs with an electrostatic coating displayed suppressed *r*_1_ relaxivity, reduced by 85% compared to their unmodified Gd-MSN counterparts, at a pH > p*K*_a_ (SI 16). The electrostatically applied polymer is able to occupy significantly more of the pore volume when compared to the (externally-grown) SI-RAFT approach, as shown by N_2_ adsorption–desorption analyses (SI 2). This allows the former applied pDMAEMA to more greatly suppress relaxivity. As such, this approach was used in all further studies.^8^ Upon exposure of these polymer-wrapped Gd-MSNs to an environment where pH < p*K*_a_, an (irreversible, see SI 16) ∼200% increase in *r*_1_ from the MRI ‘off’ state was observed due to the restoration of water exchange with bulk. As the polymer is electrostatically bound, it is able to freely diffuse into solution upon p*K*_a_ traversal, leading to the observed irreversibility.

**Fig. 2:**
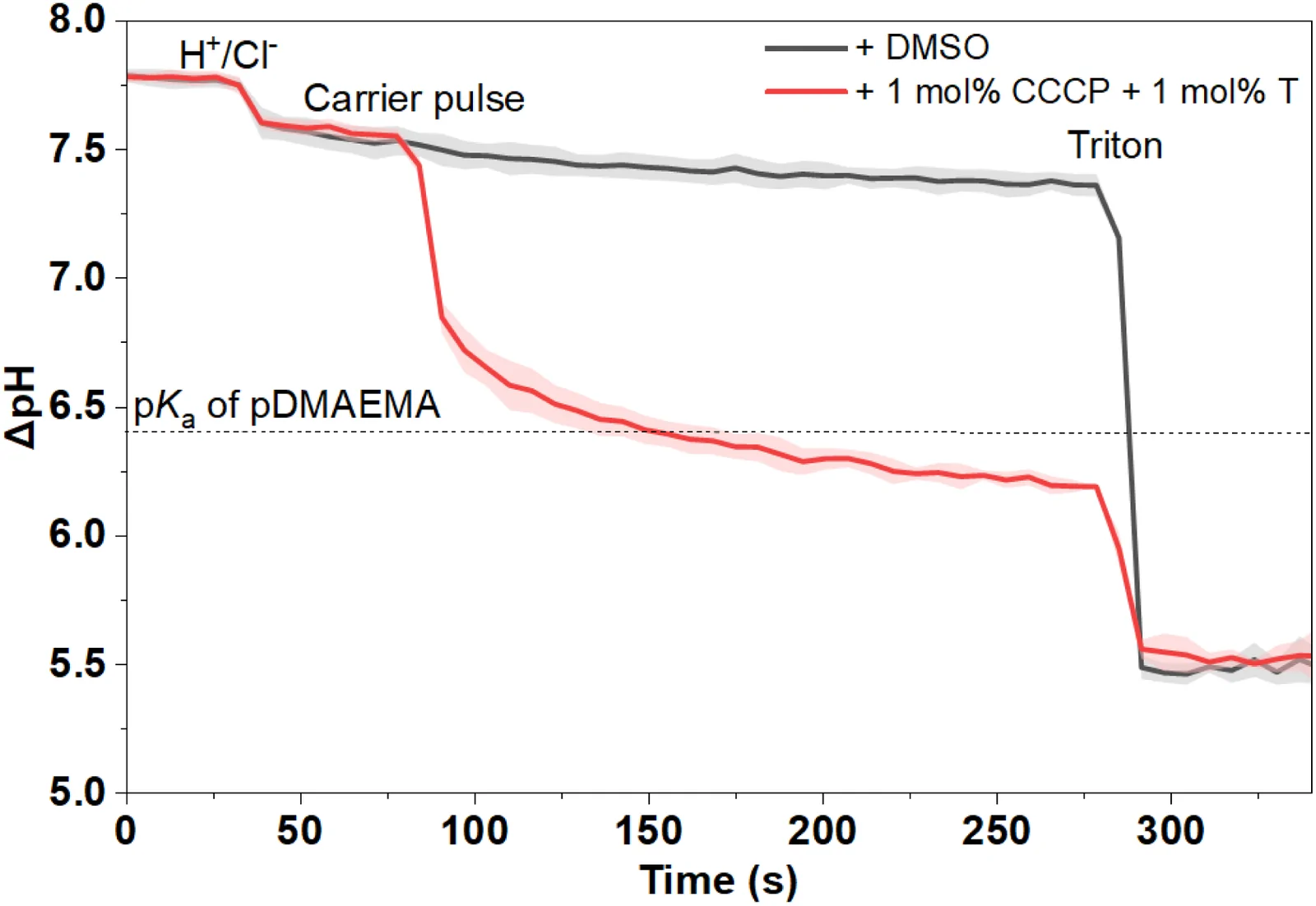
Change in intravesicular pH in large unilamellar vesicles (LUVs) containing 1 mM of the pH sensitive fluorophore HPTS, determined by ratiometric analysis of the excitation spectrum (see SI 20). Intravesicular acidification (red) is mediated by a protonophore (CCCP) and the tripodal thiourea anionophore (T, 1 mol% loading) following addition of HCl to a pH below that of the p*K*_a_ of pDMAEMA (<6.4), observations not made in the absence of ionophores (black). Triton was added at *t* = 240 s to completely lyse the vesicles for calibration. Error equal to calculated pH ± 1 s.d. (*n* = 3). For further assay conditions see SI.

**Fig. 3:**
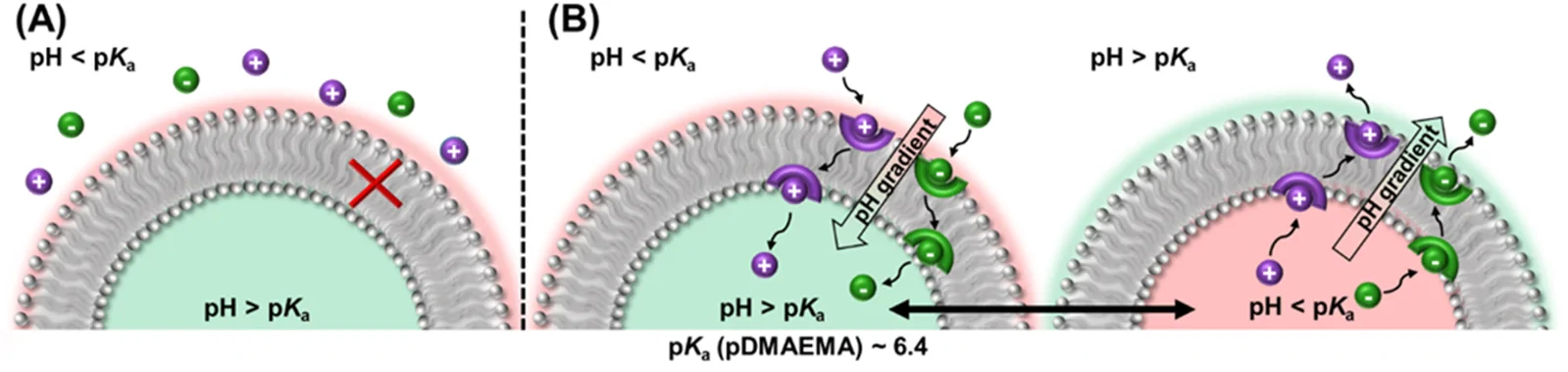
A schematic representation of intravesicular pH changes when exposed to an external pH gradient in the absence/presence of ionophores (internal pH < pDMAEMA p*K*_a_ shown red, with pH > p*K*_a_ in green). (A) In the absence of ion carriers, extravesicular ions (H^+^/Cl^−^) added to the bulk solution are unable to cross the hydrophobic lipid bilayer, leading to no pH dissipation. (B) Ionophore mediated H^+^/Cl^−^ symport enables the pH gradient to be dissipated in a reversible manner, such that when the concentration of [H^+^] is reversed, the intravesicular pH is able to re-basify.

The next step was to confirm that a transmembrane anion flux, supported by an appropriate anionophore, could be coupled to a proton flux *via* an H^+^/Cl^−^ symport mechanism such that intravesicular pH could be modulated. We investigated this with large unilamellar vesicles (LUVs) loaded with a pH-sensitive fluorophore, 8-hydroxypyrene-1,3,6-trisulfonic acid (HPTS).^40^ Cholesterol rigidified LUVs (45/55 mol% cholesterol/POPC) were prepared using a standard freeze-thaw process followed by extrusion through a 200 nm polycarbonate membrane (synthetic details SI Page 13–15) enabling extrapolation of internal pH changes to the dual-wrapped MSNs. Carbonyl cyanide-*m*-chlorophenylhydrazone (CCCP) was employed as a protonophore, and a tripodal thiourea used as a known chloride anionophore (Fig. 1).^41^ The tripodal thiourea anion carrier used within this work has established selectivity for Cl^−^ over anions such as SO_4_^2−^, PO_4_^3−^, and HCO_3_^−^, hence it was expected that cross-bilayer flux in the presence of H^+^/Cl^−^ would be more efficient than 2H^+^/SO_4_^2−^ and 3H^+^/PO_4_^3−^. This was confirmed by tracking the ratiometric fluorescence intensity (*λ*_em_ = 510 nm) of deprotonated (*λ*_ex_ = 460 nm) and protonated (*λ*_ex_ = 405 nm) HPTS in 200 nm LUVs upon exposure to HCl, H_2_SO_4_, H_3_PO_4_ and KH_2_PO_4_ (SI 18). By analysing the change in the HPTS fluorescence as a function of pH (Fig. 2, SI 19–22, using the calibration curve shown in SI 20) upon extravesicular addition of HCl followed by addition of ion carriers in DMSO, an intravesicular acidification to <pH 6.4 was resolved.^40^ Although a high cholesterol loading provides a more favourable MRI ‘off’ state, due to the associated increase in bilayer hydrophobicity (and therefore enhanced barrier to water flux), the increased membrane rigidity reduces ion carrier mobility (transport efficiency).^42,43^ This potentially unhelpful scenario was overcome by increasing ionophore loading, with optimal results obtained with the addition of 1 mol% ionophores (with respect to lipid, SI 22), and this configuration was used for subsequent relaxometric studies.^44^ In the absence of ionophores, coupled H^+^/Cl^−^ flux cannot occur, preventing dissipation of the generated pH gradient, leaving the intravesicular pH unchanged (Fig. 3(A)).

To understand whether ion transport is intrinsically coupled through the simultaneous flux of an anion and cation (symport), or the opposing flow of two similarly charged ionic species (antiport),^45^ further fluorescence studies integrating either the anionophore or the protonophore were conducted. In the presence of the CCCP protonophore alone, negligible ion flux and no significant intravesicular pH dissipation were observed (SI 22).^46^ In contrast, incorporation of solely the anionophore generated a measurable intravesicular acidification, consistent with an anionophore-mediated OH^−^/Cl^−^ antiport mechanism.^41^ The most effective and rapid dissipation of the pH gradient was achieved only when both the protonophore and anionophore were present, reflecting the coupled cationic and anionic transport dominated by an H^+^/Cl^−^ symport mechanism.

**Fig. 4:**
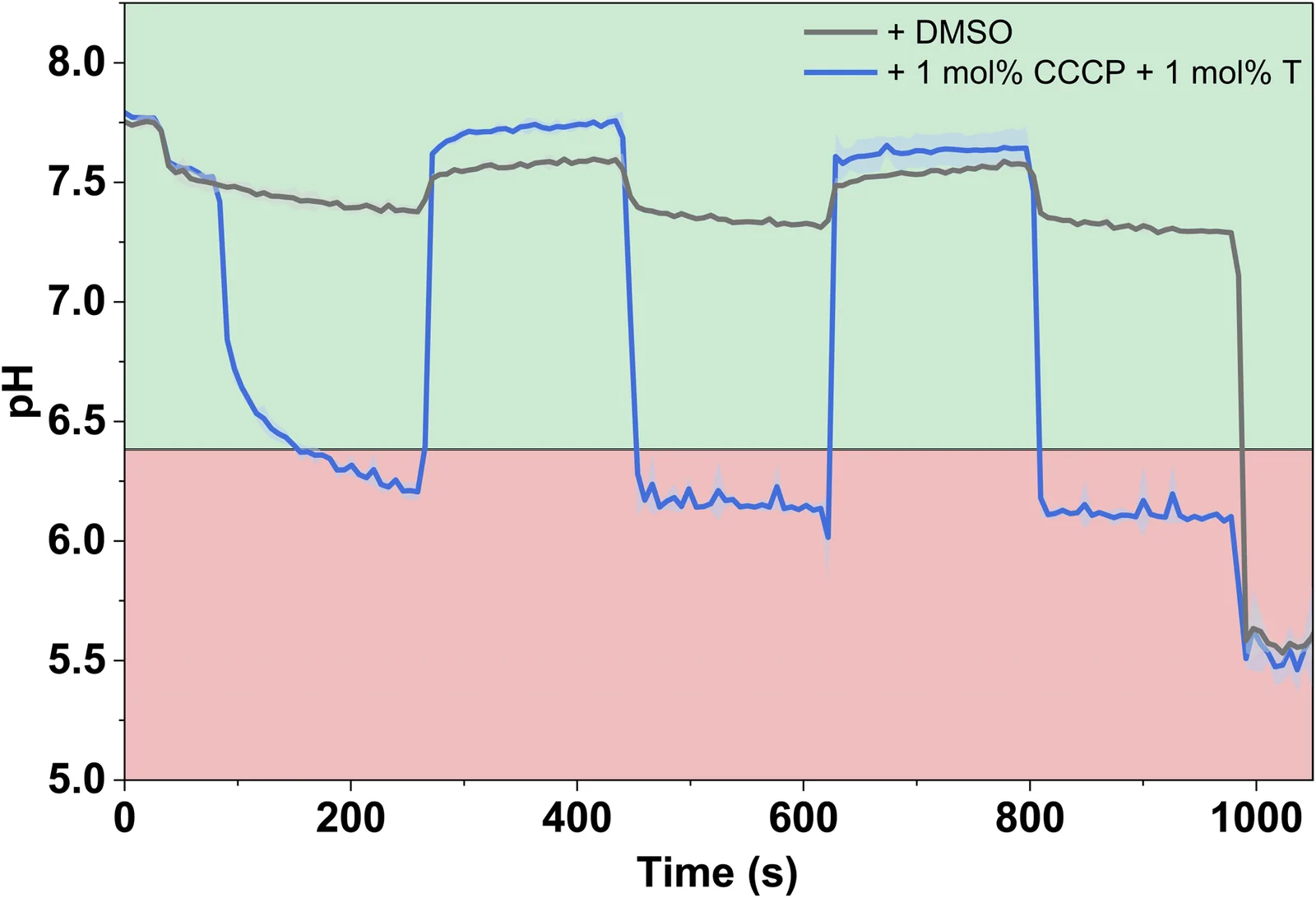
Fluorescence assay data obtained using 200 nm vesicles loaded with 1 mM HPTS, demonstrating the ability to reversibly switch intravesicular pH on repeated acid/base pulse cycling across the p*K*_a_ of pDMAEMA. HCl was added at *t* = 40 s, prior to a dual ionophore (or DMSO) pulse at *t* = 90 s. KOH was added at *t* = 270 s, and the acid/base pulses alternated. Triton X-100 was added at *t* = 990 s to lyse the vesicles for calibration. Error bars equal to calculated pH ± 1 s.d. (*n* = 3).

As the ion carriers are able to dissipate the pH gradient in either direction, as dictated by the bulk pH (Fig. 3(B)), a reversible traversal of the pDMAEMA p*K*_a_ (∼6.4) is possible within the vesicle lumen. This was confirmed by HPTS assays tracking intravesicular pH of LUVs with both ionophores present upon multiple cycles of acidification and basification (Fig. 4). Predictably, in the absence of ionophores, exposing LUVs to multiple bulk pH inversions by addition of H^+^/Cl^−^ and K^+^/OH^−^ does not significantly change the intravesicular pH (pH remains > 7.25). Given the ability to modulate internal pH in the presence of both ionophores, the addition of HCl was therefore expected to facilitate a pDMAEMA conformational change that directly improves diffusive water accessibility to pore confined Gd(iii), “switching on” contrast. These fluorescence studies on intravesicular pH, therefore, laid the groundwork from which POPC/cholesterol lipid bilayers were used to encapsulate p-Gd-MSNs.

LB-p-Gd-MSNs were prepared by encapsulating p-Gd-MSNs within pre-prepared POPC LUVs containing varying cholesterol loadings at a pH > p*K*_a_. The integrated polymer specifically responds to internal pH changes, driven by the ionophore-mediated proton-anion symport. Encapsulation was achieved through 5 sonication/vortexing cycles of 30 seconds each with a 2.5 mg mL^−1^ p-Gd-MSN suspension, before purification by centrifugation (2 × 10 300×*g*, 20 min, see SI Page 18–19 for more details). DLS measurements revealed hydrodynamic diameters of 110 ± 12 nm for p-Gd-MSNs and 128 ± 3 nm for LB-p-Gd-MSNs, consistent with the addition of a lipid bilayer.^47^ In contrast to the positively charged p-Gd-MSNs (+10 mV), membrane-wrapped p-Gd-MSNs exhibited near-neutral *ζ*-potentials, consistent with successful encapsulation by zwitterionic POPC lipids. These LB-p-Gd-MSNs were further characterised by thermogravimetric analysis (TGA) and transmission electron microscopy (TEM). TGA resolved mass losses were consistent with expectations for polymer (−16%) and polymer and bilayer (−26%) wrapped entities (for further detail and calculations see SI Page 19).

**Fig. 5:**
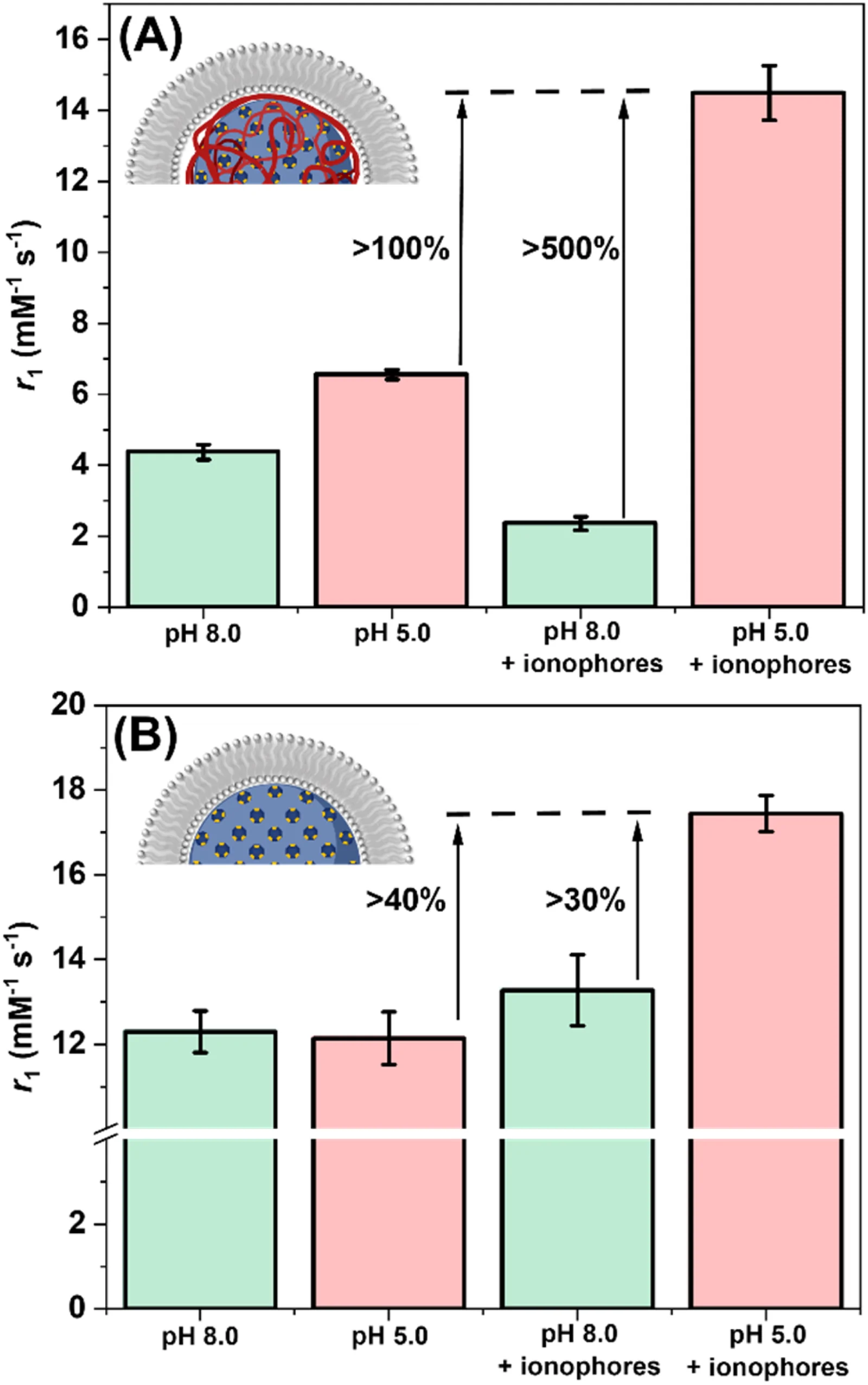
(A) pH-triggered relaxivity (1.41 T, 298 K) of LB-p-Gd-MSNs in the presence and absence of ionophores enables chloride activated proton flux. Data is shown for LB-p-Gd-MSNs (bilayer composition 45 mol% cholesterol 55 mol% POPC) at pH 5.0 and 8.0 (left), and in the presence of CCCP and tripodal thiourea (+ionophores) at 1 mol% loading with respect to lipid (right). A substantial MRI switch ‘on’ is observed in the presence of ionophores when pH is initially lowered to below the p*K*_a_ of pDMAEMA. (B) Relaxometric data of LB-Gd-MSNs (particles lacking the polymer coating) under identical conditions. The significantly attenuated MRI ‘off’ state and relaxivity switches highlight the importance of the polymer in both initial water flux suppression and in stimuli-responsiveness. Error bars obtained from a linear regression analysis on linear plots of 1/*T*_1_*versus* [Gd(iii)] to obtain a value for the gradient, which is equal to *r*_1_ ± 1 s.d.

To optimise the MRI “off” state and maximise relaxivity switching, 45 mol% cholesterol doped lipid bilayers were employed.^28^ Aligning with previous studies,^25^ cholesterol doping of the membrane within the LB-p-Gd-MSN architecture improved the MRI “off state” in the presence of H^+^/Cl^−^, pH 5.0 (*r*_1_ ∼ 6.5 mM^−1^ s^−1^ for 45 mol% cholesterol *c.f. r*_1_ ∼ 8.5 mM^−1^ s^−1^ at 1.41 T for 30 mol%) (SI 23). In the absence of ionophores, the lipid bilayer effectively suppresses the pH-response of p-Gd-MSNs as afforded by the pDMAEMA coating. Control experiments at pH < p*K*_a_ confirmed that incorporation of CCCP alone into LB-p-Gd-MSNs, relative to the native architecture, resulted in minimal relaxivity switching (22%, SI 24), verifying that proton flux must be coupled to chloride transport maintain charge electroneutrality. When the protonophore CCCP and a tripodal thiourea anionophore are integrated by external addition, and pH < p*K*_a_, H^+^/Cl^−^ symport results.^33^ This restores cross-bilayer H^+^ flux, decreasing intravesicular pH, protonating pDMAEMA, and substantially enhancing paramagnetic exchange with bulk and so *r*_1_. Specifically, for ionophore impregnated LB-p-Gd-MSNs, upon introduction of H^+^/Cl^−^, an MRI switch ‘on’ results, whereby *r*_1_ increases from 2.4 ± 0.2 mM^−1^ s^−1^ to 14.5 ± 0.8 mM^−1^ s^−1^ (Fig. 5(a), ∼500% upon initial exposure). Since the triggering proton flux is inherently coupled to an associated anion flux, the observed relaxivity switches were in line with the known anion selectivity of the tripodal thiourea carrier (Δ*r*_1_ Cl^−^ > Δ*r*_1_ SO_4_^2−^, SI 25). In the absence of the internal pH-responsive polymer, ion flux response is irreversible, smaller and mechanistically different, with *r*_1_ increasing from 12.1 ± 0.6 mM^−1^ s^−1^ to 17.4 ± 0.4 mM^−1^ s^−1^ (Fig. 5(b), ∼44%) upon transporter addition at pH 5.0.

The observed relaxivity changes were rationalised using SBM theory, which accounts for modulation of the water pool accessible to the Gd(iii) centres and changes in diffusive exchange dynamics.^48,49^ To approximate the effect of a reduced water pool, the effective inner-sphere residence time (*τ*_M_) was adjusted within the model to represent the average interval between encounters of the paramagnetic centre with unrelaxed water molecules.^50–52^ While *τ*_M_ is not physically altered by polymer or lipid bilayer encapsulation, this approximation provides framework for describing the observed pH- and ionophore-dependent relaxivity behaviour. Within this model, altering effective *τ*_M_ from 5700 ns to 1050 ns drives an inner-sphere derived change in *r*_1_ from ∼3 mM^−1^ s^−1^ to ∼13 mM^−1^ s^−1^ in line with experimentally observed values (SI 26).

In a standard 2 second inversion-recovery experiment, a native (*i.e.*, unmodified) Gd-MSN dispersion will relax about 4.6 × 10^22^ water molecules (where exchange with bulk has not been post-synthetically sterically restricted through external particle coatings). According to the BJH derived pore volume (SI 2), 1 mg of Gd-MSNs contains an estimated 5.6 × 10^19^ water molecules within the pores, and 1.6 × 10^18^ within the supported lipid bilayer interfacial space.^47^ Only this water is available to the chelates in the case where water flux is entirely limited (*i.e.*, with no access to bulk solution). This highly restricted water pool will be fully relaxed on a low ms timescale, rendering the chelates inactive (in regards to contrast generation) for the majority of the spectral acquisition period.

**Fig. 6:**
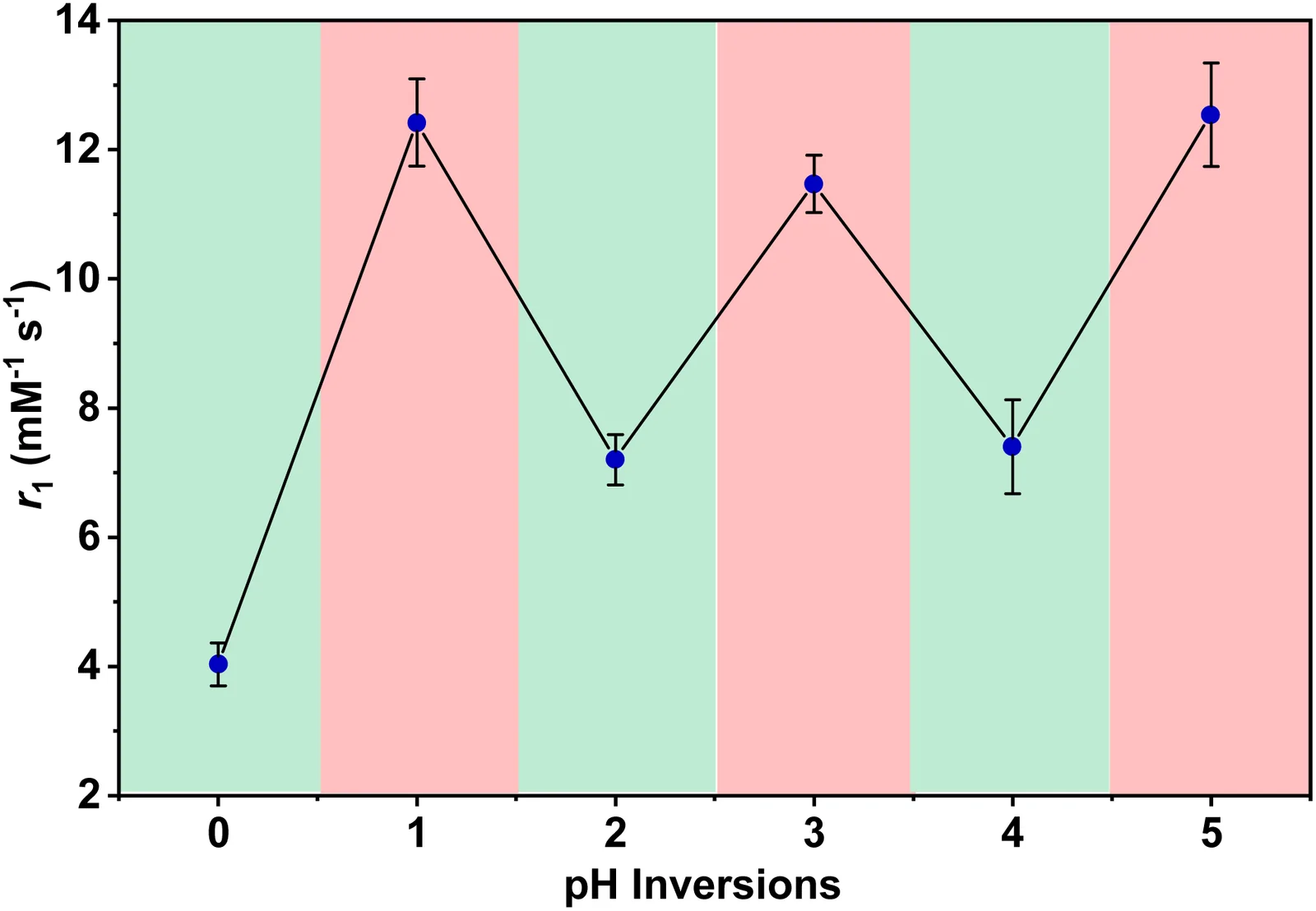
*r*_1_ values (measured at 1.41 T, 298 K) for the LB-p-Gd-MSNs containing 1 mol% CCCP and anionophore, throughout three pH reversal cycles, demonstrating a quasi-reversible *r*_1_ switch on repeated pH inversions. Error bars were obtained from a linear regression analysis on linear plots of 1/*T*_1_*versus* [Gd(iii)] to obtain a value for the gradient, which is equal to *r*_1_ ± 1 s.d.

In a significant advance on prior ion carrier modulated MRI contrast in vesicles, polymer-incorporated LB-p-Gd-MSNs incorporating both ionophores displayed a quasi-reversible relaxivity switching over three successive pH cycles (Fig. 6). This is attributed to the ionophore-mediated dissipation of the proton/anion concentration gradient in either direction, enabling reversible modulation of contrast, as elucidated by the fluorescence data obtained using HPTS containing vesicles (Fig. 4). These results reveal that the confinement of the pDMAEMA coating within the lipid bilayer prevents protonated polymer diffusion into bulk solution, unlike the non-membrane encapsulated analogue. DLS measurements corroborate these observations, revealing a quasi-reversible swelling and deswelling of the confined polymer layer (± 20 nm) in response to pH modulation (SI 28). Supramolecular interactions between the polymer and anions (*e.g.*, aqueous Cl^−^) are known to interfere with the complete recovery of the collapsed polymer upon deswelling.^53,54^ Therefore, the observed lack of complete reversibility is attributed to the strong hysteresis of pDMAEMA, whereby these solvent and ion effects strongly influence the correlation of polymer conformation to pH. The subsequent smaller, reversible changes remain well above the detectable threshold for clinical imaging, and would therefore aid in reducing off-target activation.^55^

**Fig. 7:**
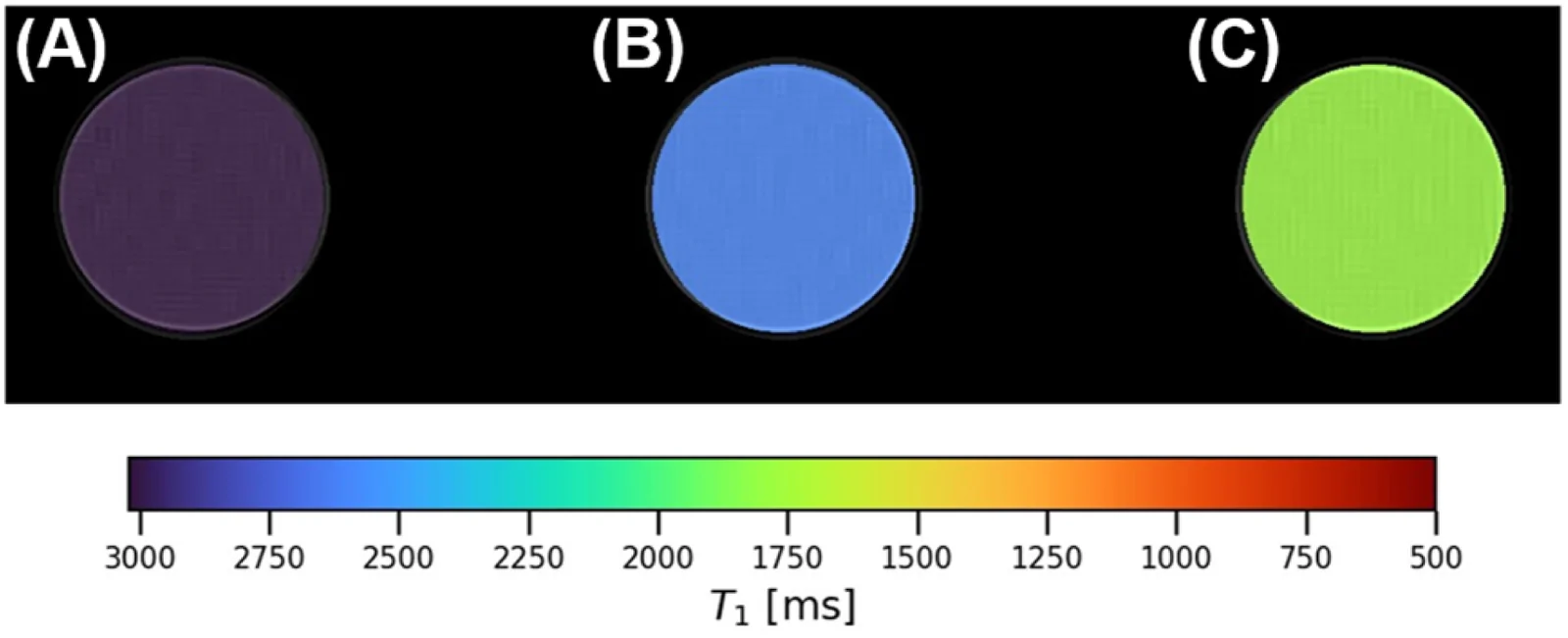
A colourised phantom MRI *T*_1_ map acquired of (A) pH 7.0 aqueous solution, (B) LB-p-Gd-MSNs measured at pH > p*K*_a_ and (C) carrier activated LB-p-Gd-MSNs measured at pH < p*K*_a_. The images were acquired at 4.7 T (298 K) and show the correlated decreases to the *T*_1_ times (3020 ± 15, 2603 ± 19, and 1178 ± 29 ms, respectively of *ca.* 0.9 mg mL^−1^ solutions). Raw MRI phantom images can be found in SI 27.

*T*_1_ weighted phantom imaging on a 4.7 T clinical MRI scanner (Fig. 7) demonstrated that significant changes in MRI contrast are clearly observed within a clinical scan for LB-p-Gd-MSNs that integrate both cationophore and anionophore. The strong contrast switch when pH traverses the p*K*_a_ of the pDMAEMA polymer highlights the translational potential of this approach to imaging disease-relevant pathology (pH < 6.4) coupled with specific ion presence.

## Conclusions

Paramagnetically doped mesoporous nanoparticles support high contrast levels, in large part, because of their native rotational correlation times. This can be heavily suppressed by coating the particle periphery with a water-blocking polymeric organic coat and suppressed further still if this construct is then additionally wrapped in a lipid bilayer membrane (generating an ameliorated MRI ‘off’ state). As the polymeric coat is conformationally pH-responsive, then so is supported MRI contrast. When the bilayer is primed to support the symport flux of specific anions, associated protons, and (by extension) molecular water, then very large switches in relaxivity are enabled in the presence of specific environmental conditions of anion presence and pH. pH-dependent imaging in the presence of a baseline chloride concentration, acting effectively by “anion-assisted” pH-responsive contrast, addresses a clear clinical need by offering a route to enhanced specificity in the detection of disease states such as tumours.^56^ The reversible nature of the contrast response further enables spatial specificity, because upon diffusion of the contrast agent away from a pathological microenvironment into healthy tissue, the particles will revert to their MRI ‘off’ state, minimising off-target signal generation. By integrating different polymer compositions (p*K*_a_ values) a library of specific microenvironment responsive-MRI probes can be designed that align with pathologies of interest.

## Author contributions

J. P. S. and A. M. D. synthesised the particles and collected experimental data. C. M. E. assisted with data collection and aided in experimental design. A. K. synthesised the anionophore. J. J. D. and M. J. L. conceptualised, designed and supervised the project. A. M. D., J. P. S., C. M. E., M. J. L and J. J. D. wrote the manuscript. All authors have given approval to the final version of the manuscript.

## Conflicts of interest

There are no conflicts to declare.

## Supplementary Material

SC-017-D6SC05306H-s001

## Data Availability

All referenced data is available in the supplementary information (SI). Supplementary information is available. See DOI: https://doi.org/10.1039/d6sc05306h.
