# Supplementary material for: Ion transport mediated pH-responsive MRI contrast in dual wrapped MSNs
Source: Chem Sci. 2026 Jul 20. Online ahead of print. doi: 10.1039/d6sc05306h (PMC13430726; doi:10.1039/d6sc05306h)
Supplement: SC-OLF-D6SC05306H-s001 [file SC-OLF-D6SC05306H-s001.pdf]

Supplementary Information (SI) for Chemical Science.
This journal is © The Royal Society of Chemistry 2026

Supplementary Information

Ion Transport Mediated pH-responsive MRI Contrast in Dual Wrapped MSNs

James P. Smith [a] ‡, Anna M. Duncan [a] ‡, Connor M. Ellis [a], Aidan Kerckhoffs [a], Matthew J. Langton* [a], Jason J. Davis* [a]

a. Department of Chemistry, University of Oxford, South Parks Road, Oxford, OX1 3QZ, UK. E-mail: Jason.Davis@chem.ox.ac.uk; Matthew.Langton@chem.ox.ac.uk

‡ These authors contributed equally.

Experimental

Materials

Triethanol amine (TEA) was purchased from Scientific Laboratory Supplies. 37% hydrochloric acid (HCl), N,N-dimethylformamide (DMF) and 70% nitric acid (HNO_3) were purchased from Fischer Scientific. DOTA-NHS ester was purchased from CheMatech. POPC was purchased from Avanti polar lipids. All other chemicals were purchased from Merck and used as received. Ultrapure water (Millipore) with a resistivity of 18.2 MΩ·cm was used throughout.

Characterisation

NMR spectra were recorded by dispersing the sample (*ca.* 5 mg) in the desired deuterated solvent and acquired on a 2-channel Bruker AVIII 400 nanobay instrument running TOPSPIN 3 equipped with a 5 mm z-gradient broadband multinuclear probe. Attenuated Total Reflectance infrared (ATR-IR) spectra were recorded on an IRTracer-100 (Shimadzu) spectrometer. Ultraviolet-visible (UV-Vis) spectra were obtained using a UV-2401PC (Shimadzu) spectrometer. TEM images were obtained by FEI Tecnai 12 Transmission Electron Microscope operated at 120 kV. Samples for TEM were prepared by incubating the copper grids (for 2 minutes) with a drop of an aqueous colloidal suspension of the nanoparticles (*ca.* 0.5 mg mL^{-1}). Inductively Coupled Plasma Mass Spectrometry (ICP-MS) measurements were performed using a PerkinElmer NexION 2000B ICP-MS spectrometer. Particle dispersions for ICP-MS were digested in 3.00 mL of concentrated (70%) HNO_3 at room temperature for > 24 h. Then, 0.95 mL of the digested solutions were taken out and adjusted to a final volume of 50 mL using ultrapure water. The [Gd^{3+}] calibration curve was prepared using SPS-SW2 standard (Spectrapure Standards, Oslo). A Malvern Zetasizer Nano with a 532 nm laser as the light source was employed for the Dynamic Light Scattering (DLS) analysis. DLS samples were prepared by dispersing the nanoparticles (*ca.* 1 mg mL^{-1}) in a potassium sulfate buffer (5 mM). Barrett–Joyner–Halenda (BJH) nitrogen adsorption-desorption analyses were obtained using a Micromeritics TriStar II PLUS surface characterisation analyser. Thermogravimetric analysis (TGA) was performed using a METTLER TOLEDO micro and ultra-micro balances. MRI measurements were performed using an inversion recovery pulse sequence on a

Spinsolve 60 T_1 Magritek 1.4 T NMR machine (2 scans, acquisition time 1.6 s, repetition time of 10 s, max inversion time of 5 s and 11 steps). The measurements were recorded for 0.5x, 0.25x and 0.125x dilutions of each sample, with the values calculated from the gradient of the linear fit (r_1) of a plot of $1/T_1$ versus the concentration of Gd (III) (as determined by ICP-MS). A Hanna Instruments pH210 Microprocessor-based Bench pH meter was used to obtain accurate pH measurements. Calibration took place using pH 4.01 and pH 7.01 buffer solutions bought from Hanna Instruments.

Synthesis of Gd-MSNs

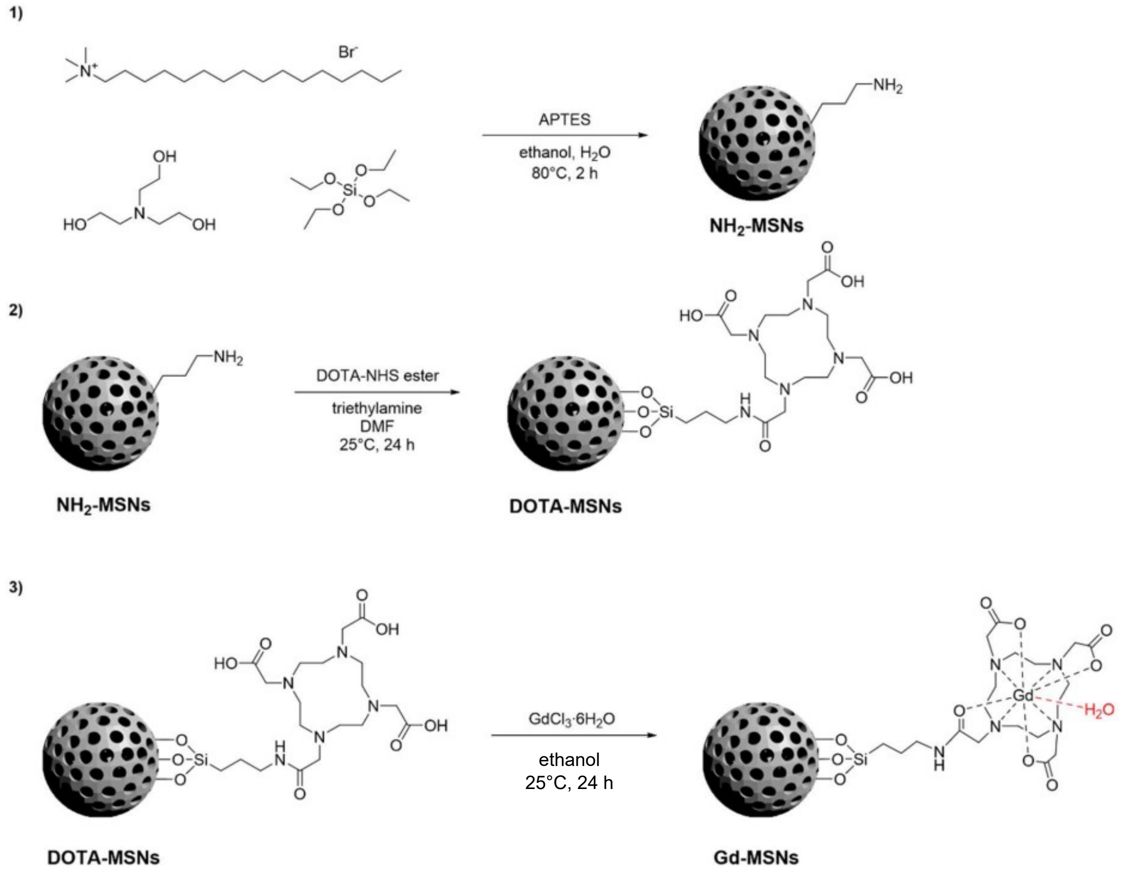

ESI 1. Schematised depiction of the synthetic steps required to produce Gd-MSNs.

Mesoporous Silica Nanoparticles Covalently Modified with Gd-DOTA (Gd-MSNs)

0.3% NH_2-MSNs: The synthesis of double-delay NH_2-MSNs was performed as a combination of the syntheses for short-delay and long-delay NH_2-MSNs previously reported by our group.[1, 2] Cetyl trimethylammonium bromide (CTAB, 0.645g, 1.77 mmol) and triethanolamine (TEOA, 1.029g, 6.9 mmol) were dissolved in 1.88 mL water and 16.20 mL ethanol. The mixture was stirred at 80 °C for 20 minutes to allow for micelle formation. Tetraethyl orthosilicate (TEOS, 1.156 mL, 5.18 mmol) was then added dropwise at 1 mL min^{-1} into the reaction mixture under stirring. After 10 minutes, 3-aminopropyltriethoxysilane (APTES, 99%, 2.6 μL) was added to the mixture. After 50 additional

minutes of stirring, another 2.6 µL of APTES was added alongside 2.23 µL of TEOS. The reaction was then stirred for a total reaction time of 2 h. The mixture was cooled down to room temperature, whereupon the particles were collected by centrifugation (10,300 x g, 20 minutes). The particles were then purified using centrifugation in ethanol (2x). 30 minutes of sonication in acidified EtOH (10 vol%) was then used to remove the CTAB template. The particles were washed two additional times with EtOH. The particles were then collected by centrifugation and dried under vacuum for 24 h to yield the desired double delay aminated MSNs.

Gd-MSNs: 200 mg of dried NH_2-MSNs were dispersed in 15 mL DMF by sonication. Then, 5.25 µmol of DOTA-NHS ester and 150 µL of triethylamine were added to the reaction flask. The resultant mixture was stirred at r.t for 24 h. The nanoparticles were collected by centrifugation at 10,300 x g for 20 minutes and washed three times with EtOH to produce the desired DOTA modified MSNs (DOTA-MSNs). The obtained DOTA-MSNs were dispersed in 10 mL EtOH to which 10 µmol of $GdCl_3.6H_2O$ was added. Then, vigorous stirring was carried out for 24 h at r.t.. The Gd-doped nanoparticles (Gd-MSNs) were washed three times with EtOH and dried under vacuum.

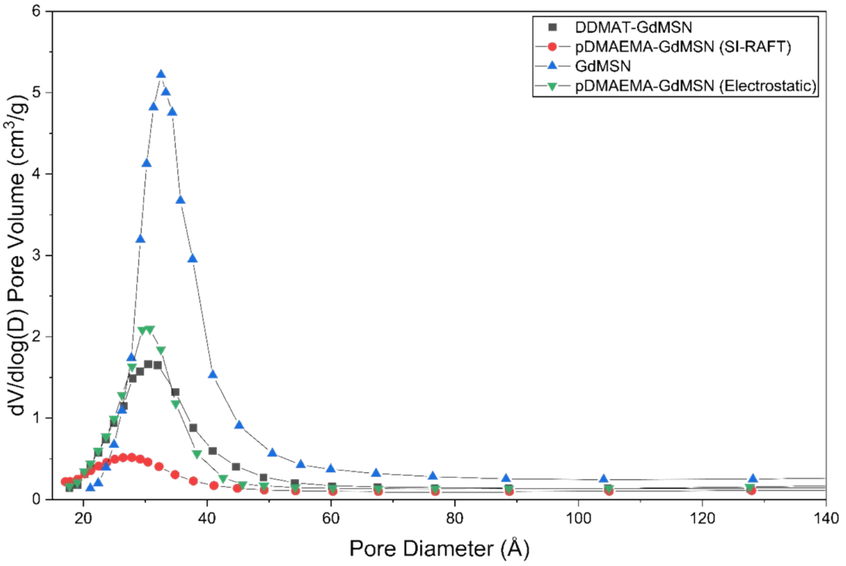

ESI 2. Barrett-Joyner-Halenda (BJH) pore size distributions for unmodified Gd-MSNs and pDMAEMA coated Gd-MSNs (pDMAEMA-Gd-MSNs). The isotherms reveal that the BJH derived pore volume decreases by 0.621 cm^3/g in the case of electrostatic polymer application (bare GdMSN and pDMAEMA-GdMSN, respectively), whereas the pore volume decreases by 0.155 cm^3/g in the case of SI-RAFT polymer application (DDMAT-GdMSN and pDMAEMA-GdMSN, respectively). There is a significantly larger pore volume decrease when the polymer is electrostatically applied, implying that it is able to partially enter the pores, directly displacing internalised water.[3] In the case of the SI-RAFT polymers, the DDMAT-silane (the moiety used to prepare particles for SI-RAFT) is too large to enter

the pores and the brush density generated from it is such that, conformationally, very little, if any, polymer is able to enter the pores

(A) (B)

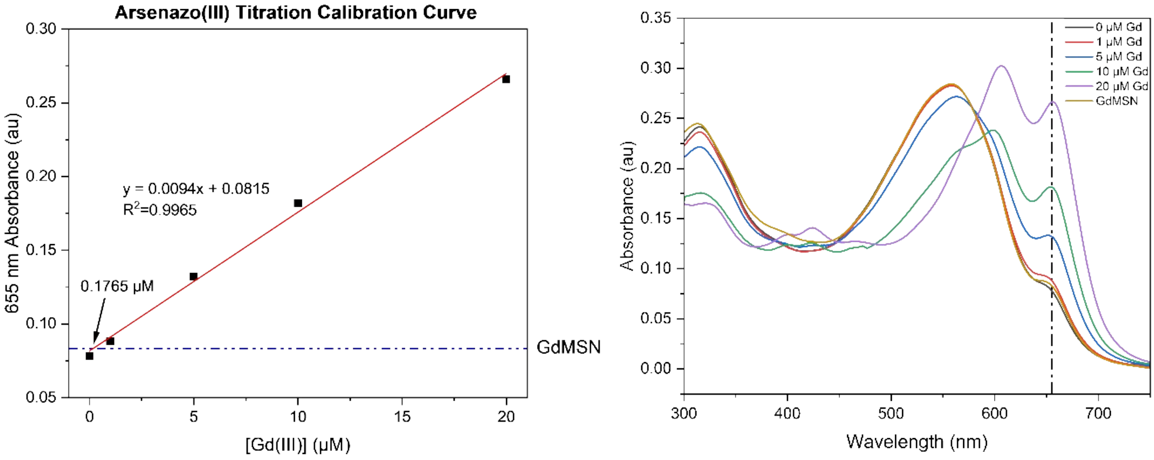

ESI 3. Determination of the concentration of unchelated Gd(III) present within the native Gd-MSNs. (A) A calibration curve of various Gd(III) concentrations using the UV-Vis absorbance of the lanthanide sensitive dye Arsenazo(III) at 655 nm. (B) Analysis of the titration revealed that there was 0.1765 μM free Gd(III) present at a concentration of 1 mg mL^{-1} of particles (0.005% of total Gd(III)).

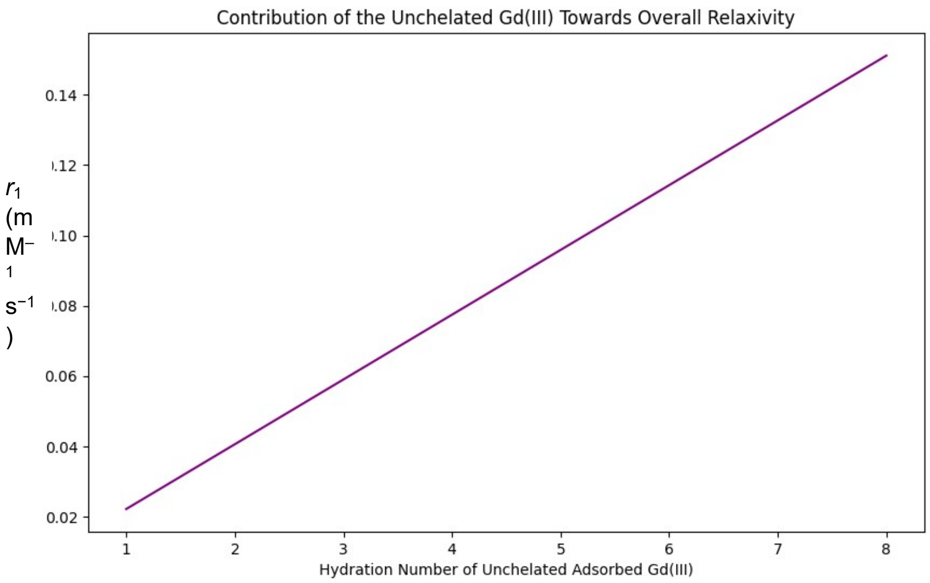

ESI 4. The contribution of the unchelated Gd(III) atoms to overall relaxivity as determined by the Arsenazo(III) titration. As the hydration number of the free adsorbed Gd(III) is not known, all possible values were examined. In the worst case scenario of q = 8, the free Gd(III) only contributes 0.14 $mM^{-1}s^{-1}$ to the total relaxivity of 27.16 $mM^{-1}s^{-1}$ (< 0.5% contribution to total relaxivity).

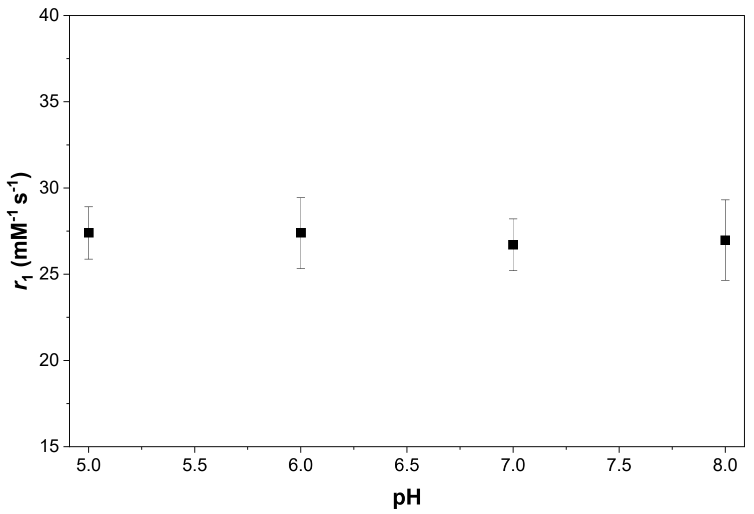

ESI 5. Longitudinal relaxivity (r_1) values (1.41 T, 298 K) of unmodified Gd-MSNs in 5 mM K2SO4 solution buffered (10 mM HEPES) to pH 5.0 (acidified with 0.01 M HCl), 6.0 (0.01 M HCl), 7.0 (basified with 0.01 M NaOH) and 8.0 (0.01 M NaOH). r_1 values are all within error and show no significant changes across the entire pH range.

Synthesis of poly(dimethyl aminoethyl methacrylate) (pDMAEMA)

The monomer (DMAEMA), chain transfer agent (4-Cyano-4-(phenylcarbonothioylthio)pentanoic acid) (CPADB), and the initiator azobisisobutyronitrile (AIBN) were dissolved in 1.5 mL THF in a 50:1:0.1 molar ratio. The solution was sealed in a glass microwave reactor vial. The mixture was degassed under argon for 15 minutes. The reaction mixture was stirred at 70 °C for 24 h, where it was then quenched by exposure to air and cooled to room temperature. The mixture was transferred to a centrifuge vial and precipitated with excess *n*-hexane. The crude product was collected by centrifugation (10,300 x g 20 minutes). To purify, the collected solid was redissolved in a minimum volume of THF, and precipitated with *n*-hexane 3 times. The product was collected and dried under vacuum for 24 hours to yield the desired polymer. The degree of polymerisation, $D_p = 45.995$ (7.2 kDa).

Synthesis of Electrostatically Coated pDMAEMA Coated Gd-MSNs (p-Gd-MSNs)

20 mg of dried Gd-MSNs were dispersed in 2 mL of THF by sonication. A solution of the previously synthesised polymer (THF, 5 mg mL^{-1}, 2 mL) was then added to the mixture. The solution was sonicated for 10 minutes, and stirred vigorously for 30 minutes. The particles were collected by centrifugation, and the coating procedure repeated one additional time. The particles were collected and dried under vacuum for 18 h to yield the desired p-Gd-MSNs.

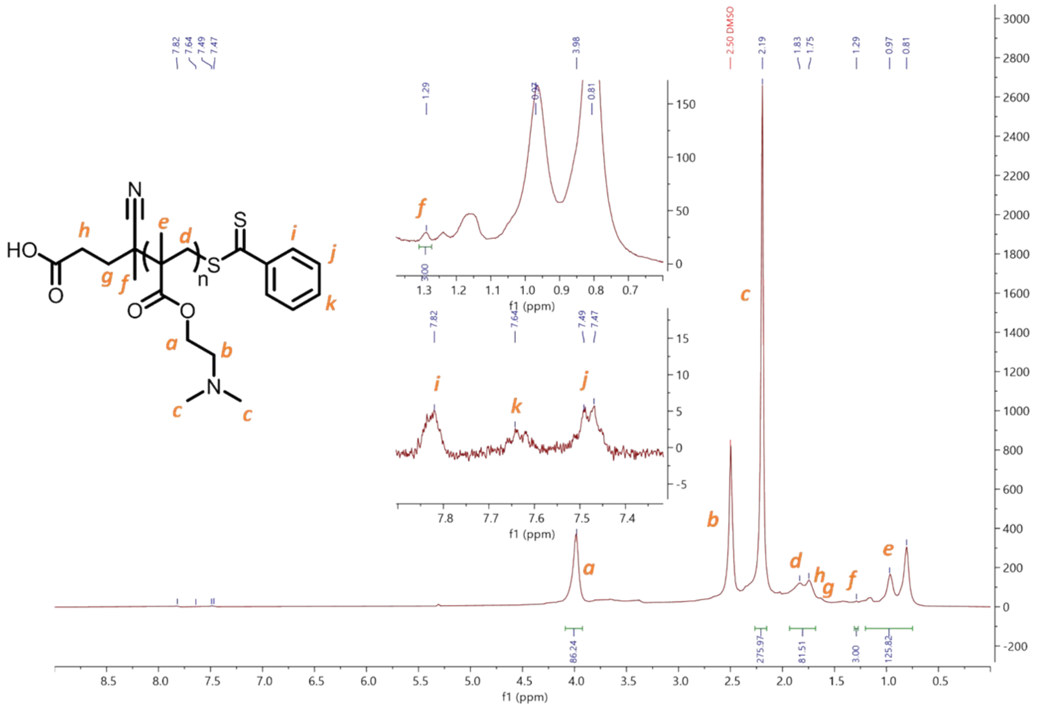

ESI 6. ^{1}H spectrum of pDMAEMA synthesised via RAFT with a theoretical D_p of 50 (The molar ratio of monomer:CTA is 50:1). Comparing the integral of ***f*** (3H, 1.29 ppm) to ***c*** (6H per repeating unit, 2.19 ppm) yields a D_p of 45.995. Integral of ***b*** is not included as it overlaps with the residual signal of non-deuterated DMSO.

^{1}H NMR (400 MHz, DMSO) δ 7.82, 7.64, 7.49, 7.47, 3.98, 2.50, 2.19, 1.83, 1.75, 1.29, 0.97, 0.81.

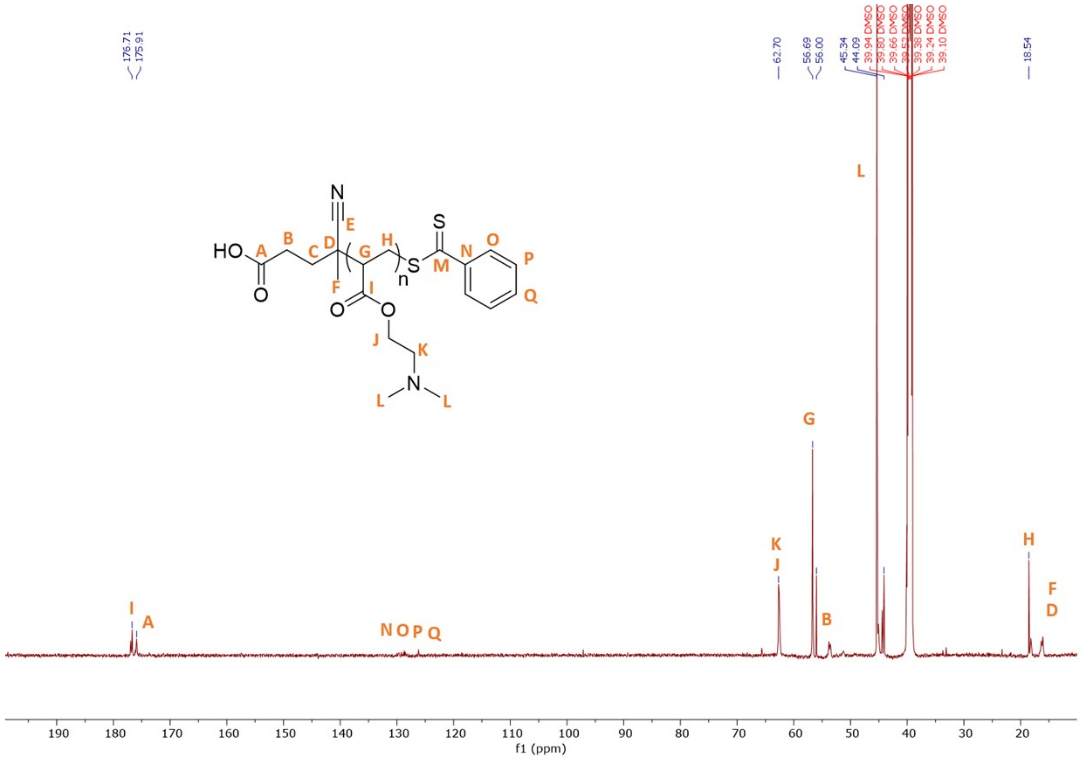

ESI 7. ^{13}C NMR (151 MHz, DMSO) δ 176.84 , 175.91, 62.63, 56.72, 53.68, 45.33, 45.05, 18.54, 16.22.

Synthesis of DDMAT-Silane

2-(Dodecylthiocarbonothioylthio)-2-methylpropionic acid (DDMAT, 600 mg, 1.65 mmol) was initially dissolved in DCM (50 mL). Then, N-(3-dimethylaminopropyl)-N'-ethylcarbodiimide hydrochloride (EDC) (315 mg, 1.65 mmol) was dissolved in anhydrous DCM (10 mL) and subsequently added dropwise to the DDMAT solution at 0 °C. The mixture was stirred for a further 10 minutes before adding (3-aminopropyl) triethoxysilane (APTES) (385 µL, 1.65 mmol) dropwise to the flask. The reaction mixture was stirred at 0 °C for 2 h, followed by further stirring at r.t. for 4 h. The crude product was concentrated under reduced pressure, and purified by silica gel column chromatography (1:2 v/v ethyl acetate/ hexane) to yield a viscous yellow oil**.** HR ESI-MS found *m/z* 590.2798, calc. *m/z* 590.2864 $[M+Na]^+$.

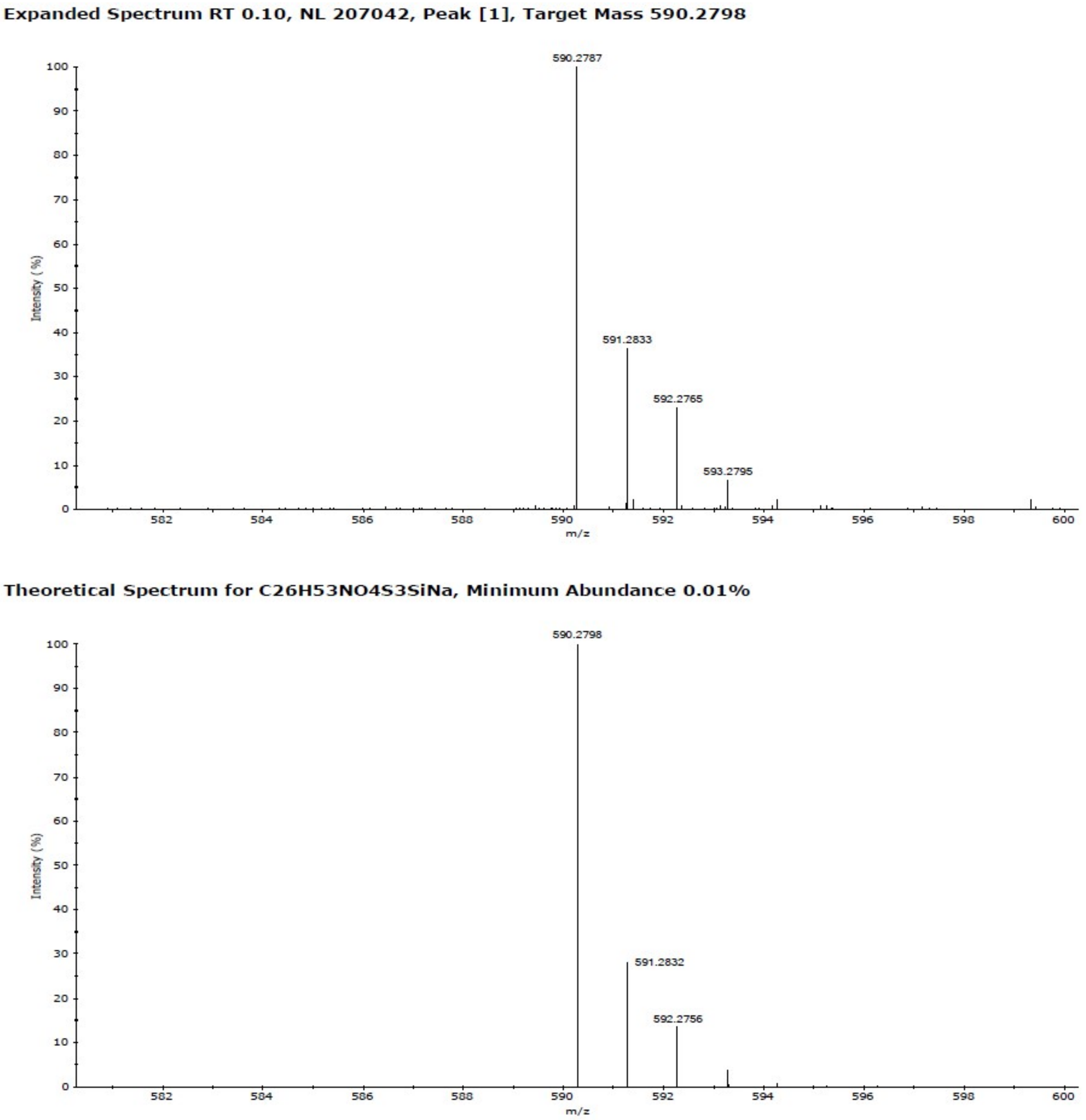

ESI 8.1. Expanded high-resolution ESI-MS of the synthesised DDMAT-Silane in MeCN, positive mode.

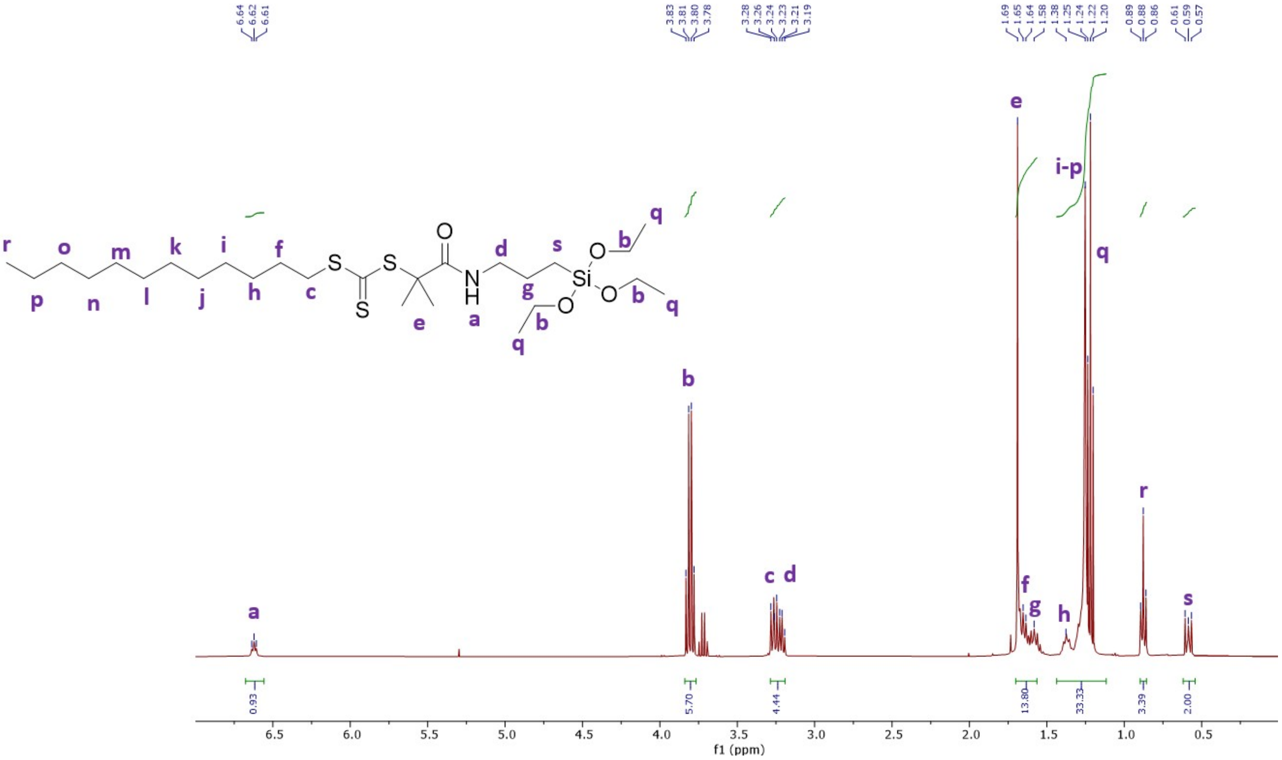

ESI 8.2. ^{1}H NMR (400 MHz, $CDCl_3$), δ 6.62 (t, *J* = 5.9 Hz), 3.81 (q, *J* = 7.0 Hz), 3.39–3.08 (m), 1.69 (s), 1.38 (s), 1.34–1.08 (m), 0.88 (t, *J* = 6.1 Hz), 0.59 (t).

Synthesis of pDMAEMA-Gd-MSNs (via SI-RAFT)

Dried native Gd-MSNs (100 mg) were dispersed in an aqueous ethanol solution (v(EtOH)/ v(H_2O) = 10 mL/ 5 mL), with ammonia (800 µL) then added into the dispersion, followed by addition of DDMAT-silane (100 µL). The dispersion was stirred at r.t. for 24 hours, followed by an ethanol wash, a DMF wash and a subsequent ethanol wash. The washed nanoparticles were collected by centrifugation (10,300 x g, 20 minutes) and then dried under vacuum. 50 mg of these particles were dispersed in DMF (9.490 mL), with the subsequent addition of 2,2′-azobis(2-methylpropionitrile) (AIBN, 0.99 mg, 6 µmol). Before degassing with argon for 15 minutes, 2-(dimethylamino)ethyl methacrylate (DMAEMA, 505 µL) was added to the mixture. The fully degassed dispersions, contained in sealed reactors, were vigorously stirred at 70 °C for 24 h. The reactions were quenched by exposing to air and subsequently cooled to room temperature, with the particles then collected by centrifugation (10,300 x g, 20 minutes). The resultant nanoparticles were re-dispersed and washed two times with DMF and one time with ethanol to remove any unreacted reagents. Finally, the obtained yellow solid was dried *in vacuo* overnight for further use.

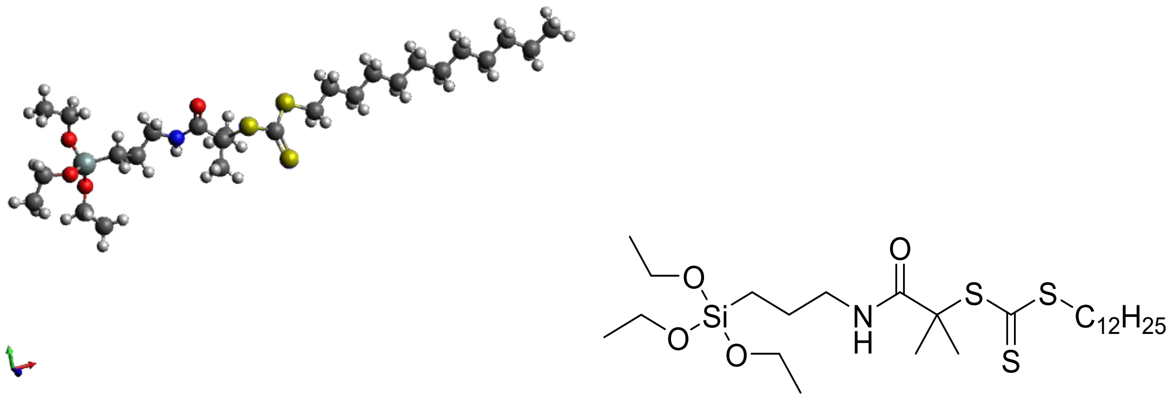

ESI 9. Density Functional Theory (DFT) employing the commonly used B3LYP (6-31G*) basis set was used to calculate the optimised geometrical structure of the DDMAT-Silane, resolving a molecular diameter of 3.21 nm. This optimised diameter is larger than the BET-derived pore size, meaning it is not able to enter the mesopores.

(A) (B)

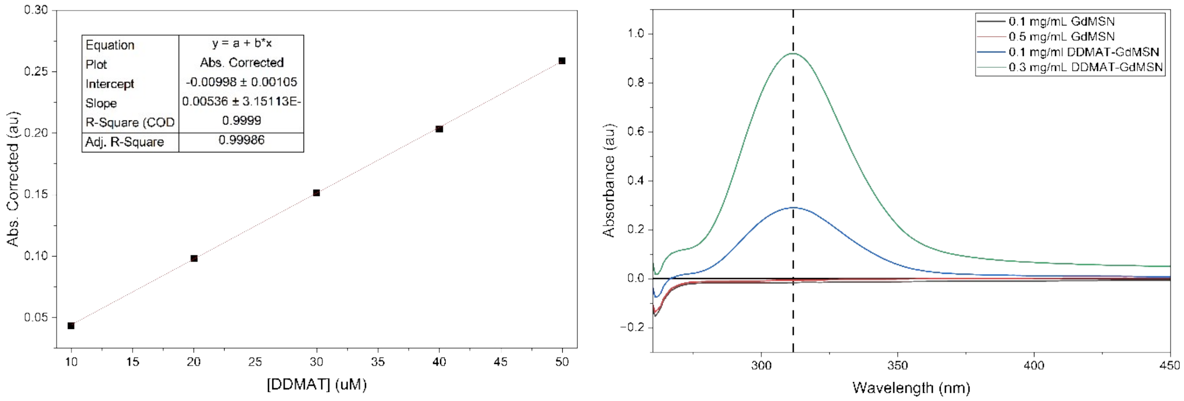

ESI 10. (A) The calibration curve obtained from the max UV-Vis absorbance of the trithiocarbonate group at 311 nm. (B) The UV-Vis spectra of unmodified Gd-MSNs and DDMAT-Gd-MSNs at varying concentrations in DMF. The absorbance at 311 nm can be used to extrapolate the grafting density of the DDMAT-silane via a previously employed literature method.[1] Grafting density of DDMAT was determined to be 3.06 groups nm^{-2}.

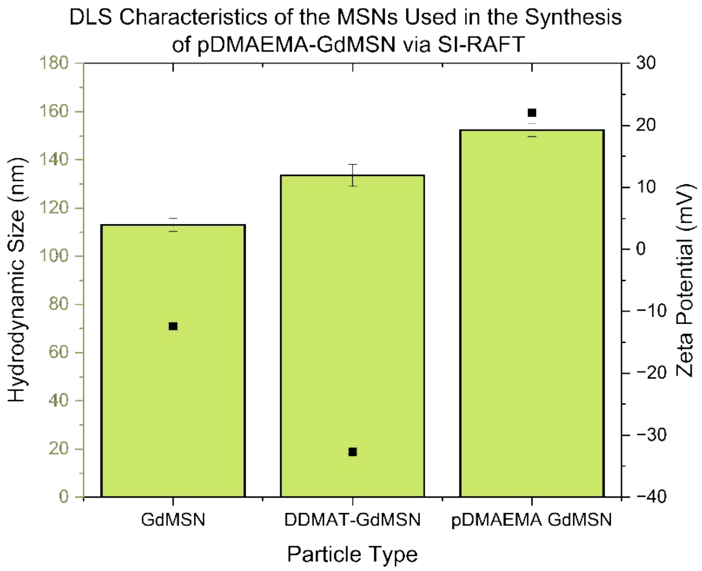

ESI 11. DLS data for the native Gd-MSNs, (DDMAT-Gd-MSNs and resultant SI-RAFT pDMAEMA-Gd-MSNs measured as a 1 mg mL^{-1} solution in pH 6.0 H_2O (+HCl). The SI-RAFT pDMAEMA-Gd-MSNs display a positively charged ζ-potential compared to a typical aminated MSN, as well as an appropriate size to undergo encapsulation within 200 nm LUVs. The error bars are reported as ± 1 s.d. either side of the mean value from three repeat measurements.

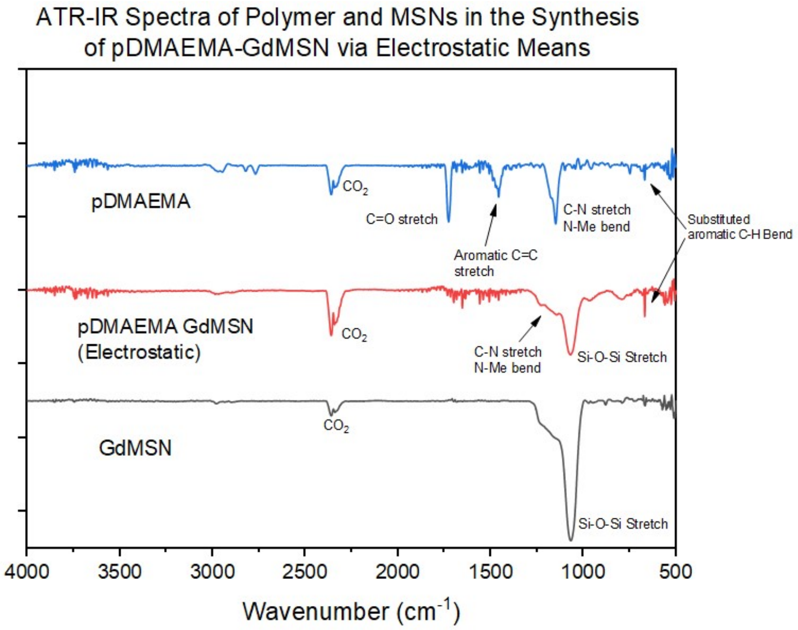

ESI 12. ATR-IR spectra for unmodified Gd-MSNs, pDMAEMA and pDMAEMA coated Gd-MSNs (electrostatic association). Silencing of characteristic pDMAEMA peaks in the electrostatically coated particle is due to conformation changes and bond motion restriction due to the tight association with the rigid scaffold. Inclusion of polymer notably increases the adsorption of atmospheric CO_2 (2300 cm^{-1}).

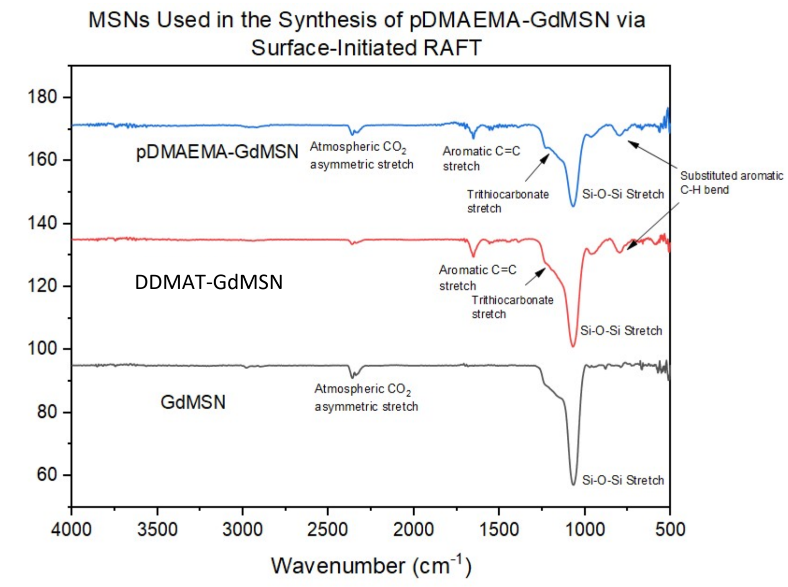

ESI 13. ATR-IR spectra acquired for unmodified Gd-MSNs, DDMAT-Gd-MSNs and pDMAEMA-Gd-MSN formed by SI-RAFT and are, as such, covalently grafted to the Gd-MSN surface.

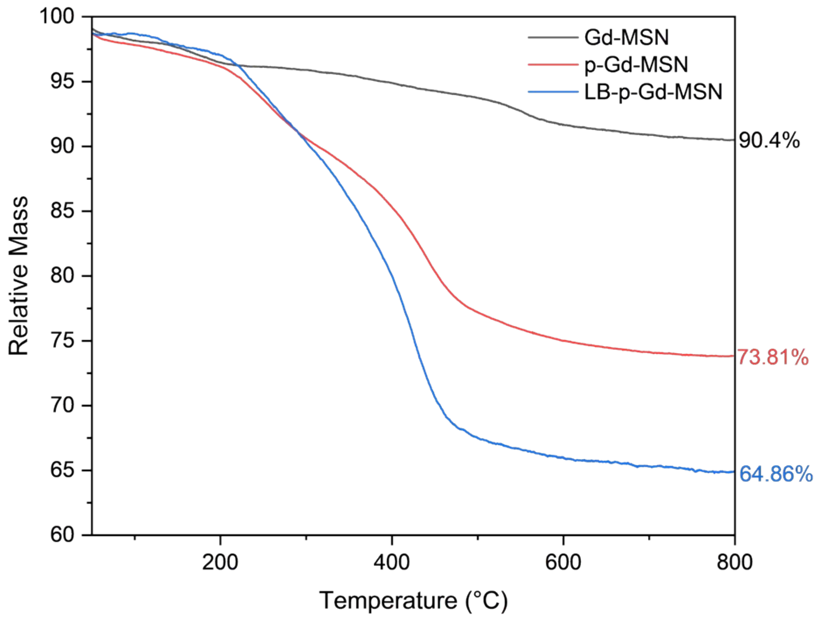

ESI 14. TGA data displaying relative mass lost during temperature ramping. A significant increase in mass lost is observed for the LB-p-Gd-MSNs compared with p-Gd-MSNs.

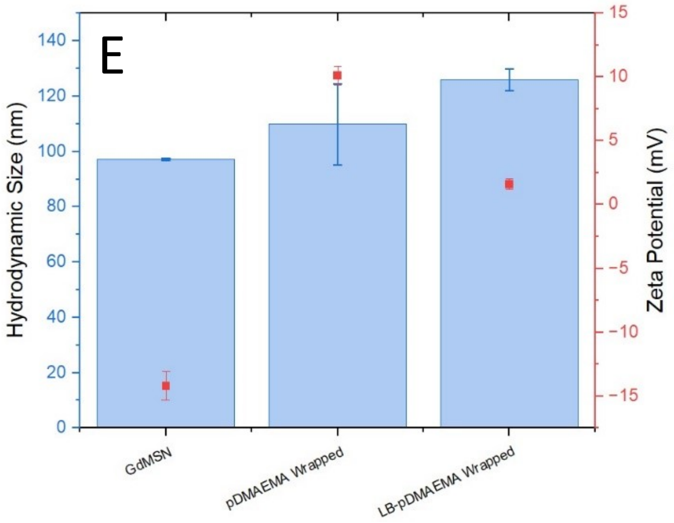

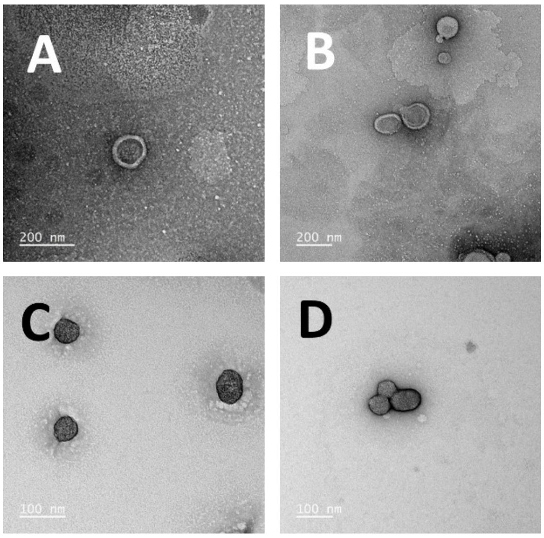

ESI 15. TEM showing (A, B) the LB-p-Gd-MSNs, (C) p-Gd-MSNs formed by SI-RAFT (D) p-Gd-MSNs formed by electrostatic coating. All samples were negatively stained with uranyl acetate. (E) DLS data for Gd-MSNs, electrostatically wrapped p-Gd-MSNs, and the LB-p-Gd-MSNs. p-Gd-MSNs demonstrate charge reversal when compared to a typical Gd-MSN, as well as an appropriate size to undergo encapsulation. The LB-p-Gd-MSNs show the associated increase in hydrodynamic size, and masking of the charged polymer through zeta reversal. The error bars are ± 1 s.d. either side of the mean value from three repeat measurements.

A (B)

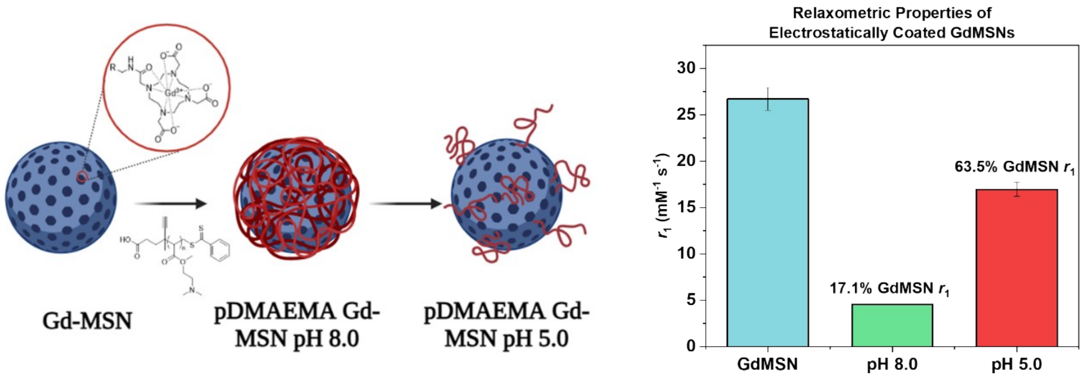

ES
I 16. (A) Schematised depiction of pH-responsive electrostatic polymer wrapping of Gd-MSNs to form p-Gd-MSNs, with a tightly bound polymer coating observed at pH 8.0 (pH > pK_a), with desorption occurring at pH 5.0 (pH < pK_a). (B) The resultant switches in longitudinal relaxivity are displayed for pH > pK_a and pH < pK_a (1.41 T, 298 K).

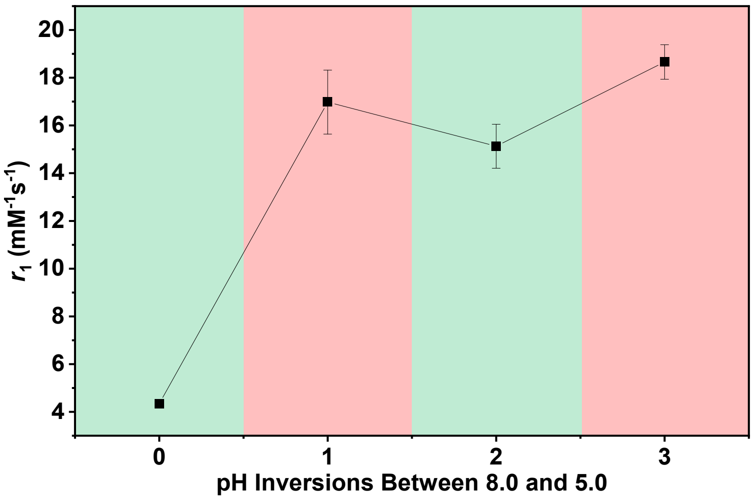

ESI 17. The irreversibility of the electrostatically coated p-Gd-MSN in longitudinal relaxivity is demonstrated over 3 pH switching cycles (1.41 T, 298 K). This particle shows minimal reversibility of relaxivity changes in response to pH, because upon acidification, the polymer is able to disperse into solution. A portion of the electrostatically associated pDMAEMA remained associated to the particle, even at pH 8.0, fouling it. Therefore, the relaxivity does not return to the optimal value of the unmodified Gd-MSN.

HPTS Transport Assays

Fluorescence assays were employed using LUVs to investigate the efficacy by which an external acidic environment, and ionophore presence, enables the internal environment within a lipid bilayer to also have its pH lowered upon addition of specific ions. The method is taken from established literature protocols, and adapted to match the conditions used in the MRI relaxivity experiments.[4, 5]

Anion Selectivity Studies

Fluorescence assays were employed using LUVs to investigate the efficiency of anion transfer in the presence of different biologically relevant species by tracking the change in I_{460}/I_{405}, which reports on the protonated/deprotonated states of 8-Hydroxypyrene-1,3,6-trisulfonate (protonated form, λ_{ex1}=405 nm, λ_{em}=510 nm; deprotonated form, λ_{ex2}=460 nm, λ_{em}=510 nm) which correlates with internal pH change following exposure to different ion pulses. 45 mol% cholesterol 55 mol% POPC films were hydrated with 1 mL 10 mM HEPES, 5 mM K_2SO_4, 1 mM HPTS. The lipid suspension was then subjected to 5 freeze-thaw cycles using liquid nitrogen and a water bath (40°C) followed by extrusion 19 times through a polycarbonate membrane (pore size 200 nm). Extra-vesicular components were removed by size exclusion chromatography on a Sephadex G-25 column eluted with buffer (10 mM HEPES, 5 mM K_2SO_4, pH 8.0, loading onto column with 2.5 mL, collection with 3.5 mL). Final conditions: LUVs (2.5 mM lipid); inside 10 mM HEPES, 5 mM K_2SO_4, 1 mM HPTS, pH 8.0; outside: 10 mM HEPES, 5 mM K_2SO_4, pH 8.0. Similar to the previous experiments, the LUVs containing HPTS (25 μL, final lipid concentration 31.35 μM) were added to buffer (1947.5 μL of 10 mM HEPES, 5 mM K_2SO_4, pH 8.0) at 25 °C under gentle stirring. A pulse of HX (17.5 μL solution, with the conditions listed out below) was added at 40 s. At 90 s, a 10 μL DMSO solution (either blank, or including mobile ion carriers at varying mol%) was added. At 240 s, detergent (25 μL of Triton X-100 in 7:1 (v/v) H_2O-DMSO) was added to lyse the vesicles and allow for normalisation of the data. Fluorescence emission intensity was monitored at λ_{em} = 510 nm (λ_{ex} = 405/460 nm). $R_t = \frac{I_{460}}{I_{405}}$ at time t was calculated as the ratio of intensities at 460 nm / 405 nm excitation. Each experimental test was repeated two times and averaged, whereby the shaded region within the plot represents ± 1 standard deviation.

1. 1 M HCl pulse (40 s); 10 μL DMSO (90 s) – green trace
2. 1 M HCl pulse (40 s); 10 μL comprised of 1 mol% T AND 1 mol% CCCP (5 μL of concentration 0.125 mM T in DMSO AND 5 μL of concentration 0.125 mM CCCP, final concentration of 0.3125 μM of each) (90 s) – black trace
3. 0.5 M H_2SO_4 pulse (40 s); 1 mol% both ion carriers, as above (90s) – red trace
4. 1 M H_3PO_4 pulse (40 s); 1 mol% both ion carriers, as above (90s) – blue trace
5. 1 M KH_2PO_4 pulse (40 s); 1 mol% both ion carriers, as above (90s) – purple trace

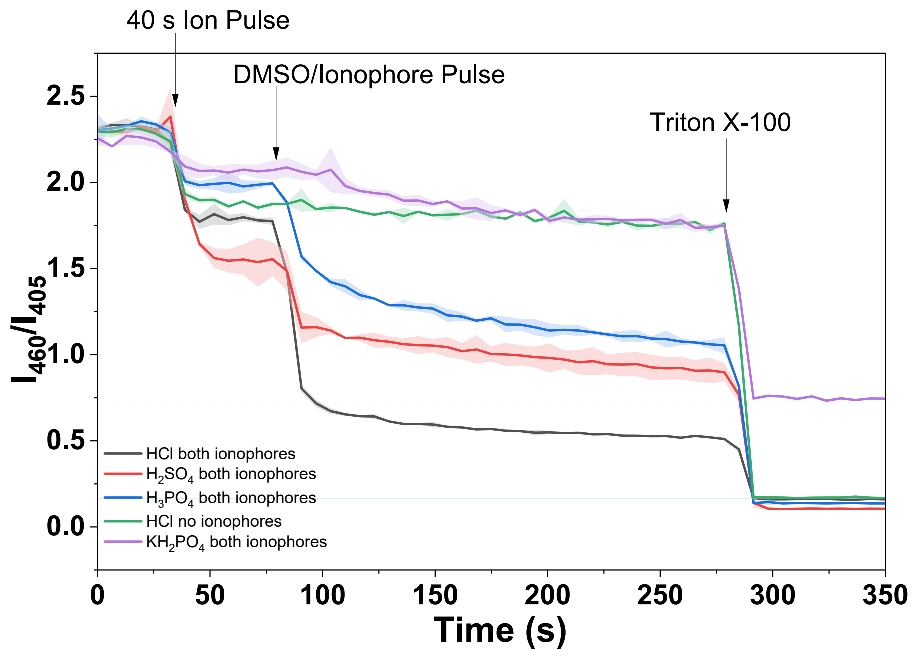

ESI 18. HPTS fluorescence assay data showing the change in fractional fluorescence intensity upon the incorporation of both ionophores (or DMSO control) followed by external acidification in specific ion presence (*e.g.,* HCl, H_2SO_4, H_3PO_4 as specified above). The data allows anion preference to be evaluated by observing the magnitude of change in I_{460}/I_{405} between 90 and 290 s. It can clearly be observed that $Cl^- > SO_4^{2-} > PO_4^{3-}$, aligning with the known selectivity of the anionophore.

HPTS Transport Assays to Quantify Intravesicular pH Change

Protocol

A thin film of lipids (45 mol% cholesterol 55 mol% POPC) was formed by evaporation of chloroform solutions on a rotary evaporator (20 °C) and then dried under high vacuum (<1.0 mbar) for at least 6 hours. The lipid film was hydrated by vortexing with the prepared buffer (10 mM HEPES, 5 mM K_2SO_4, 1 mM 8-hydroxypyrene-1,3,6-trisulfonic acid trisodium salt (HPTS), 0.5 mM KCl, pH 8.0). The lipid suspension was then subjected to 5 freeze-thaw cycles using liquid nitrogen and a water bath (40°C) followed by extrusion 19 times through a polycarbonate membrane (pore size 200 nm). Extra-vesicular components were removed by size exclusion chromatography on a Sephadex G-25 column eluted with buffer (10 mM HEPES, 5 mM K_2SO_4, 0.5 mM KCl, pH 8.0, loading onto column with 2.5 mL, collection with 3.5 mL). Final conditions: LUVs (2.5 mM lipid); inside 10 mM HEPES, 5 mM K_2SO_4, 1 mM HPTS, 0.5 mM KCl, pH 8.0; outside: 10 mM HEPES, 5 mM K_2SO_4, 0.5 mM KCl, pH 8.0.

In a typical experiment, the LUVs containing HPTS (25 µL, final lipid concentration 31.35 µM) were added to buffer (1947.5 µL of 10 mM HEPES, 5 mM K_2SO_4, 0.5 mM KCl, pH 8.0) at 25 °C under gentle stirring. A pulse of HCl (17.5 µL of 1 M solution) was added at 40 s. At 90 s, a 10 µL DMSO solution (either blank, or including mobile ion carriers at varying mol%) was added. At 240 s, detergent

(25 µL of Triton X-100 in 7:1 (v/v) H_2O-DMSO) was added to lyse the vesicles and allow for normalisation of the data. Fluorescence emission intensity was monitored at $\lambda_{em} = 510$ nm ($\lambda_{ex} = 405/460$ nm). $R_t = \frac{I_{460}}{I_{405}}$ at time t was calculated as the ratio of intensities at 460 nm / 405 nm excitation. Each experimental test was repeated at least three times and averaged, whereby the shaded region within the plot represents ± 1 standard deviation. In order to calibrate the HPTS assay for these particular conditions using vesicles, the ratiometric fluorescence response of HPTS was recorded at a range of pHs. 1 mol% CCCP and 1 mol% tripodal thiourea in DMSO (w.r.t. POPC) was added to equilibrate the pH inside the vesicles with the bulk solution pH. After the addition of triton, the fluorescence ratio was recorded, and then plotted as a function of pH, in order to obtain a calibration curve from which R_t can be quantitatively converted to absolute pH values using the following equations.

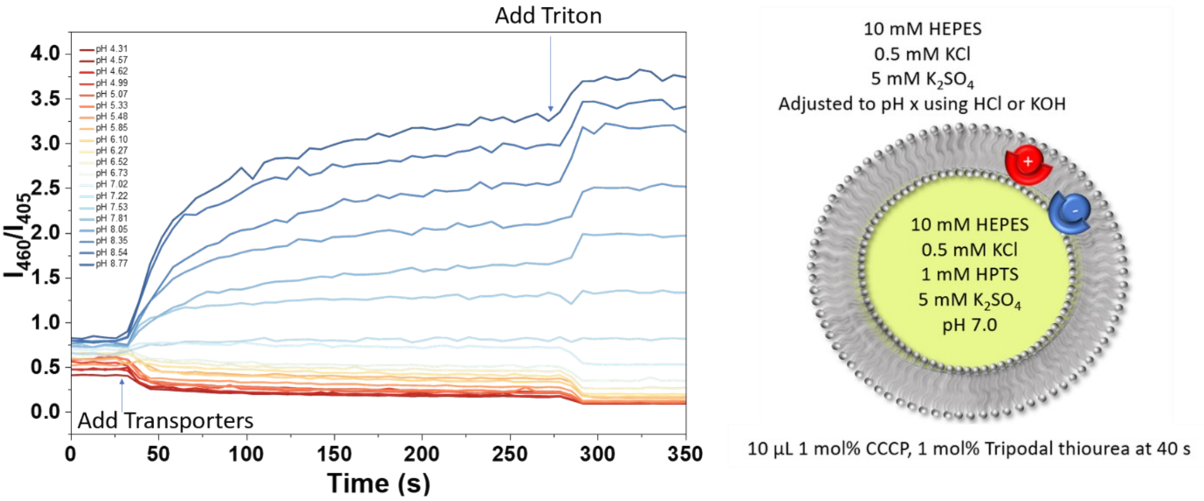

ESI 19. Plotted I_{460}/I_{405} HPTS assay trace against time, enabling calibration for the vesicles of the particular experimental conditions used in this study. The intra- and extra-vesicular conditions utilised are schematised.

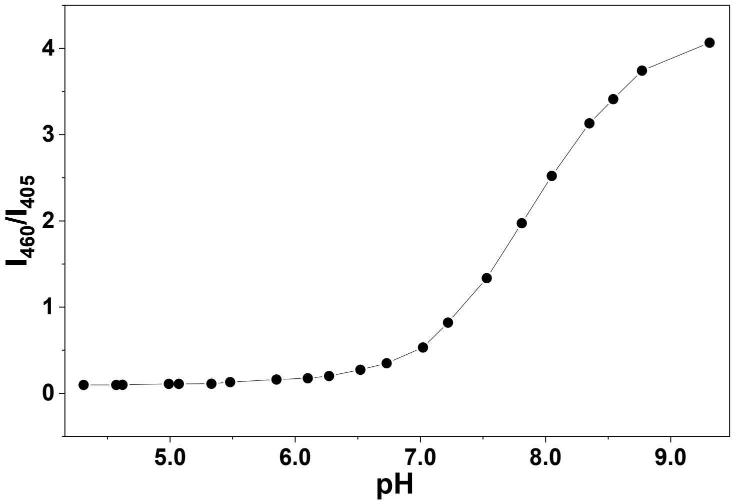

ESI 20. Plot of fractional fluorescence intensity (I_{460}/I_{405}) as a function of pH of external buffer solution utilised, taken from ESI 19.

$$K_a = \frac{[H^+][A^-]}{[HA]}$$

$$pH = pK_a + log\frac{[A^-]}{[HA]}$$

$$y = d + log\frac{ax - b}{c - x}$$

Where $x = \frac{I_{460}}{I_{405}}$, $y = pH$

$$pH = d + log\frac{ax - b}{c - x}$$

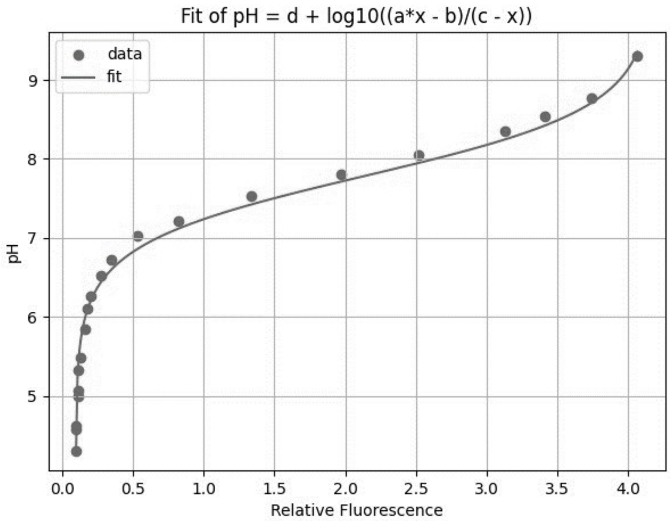

ESI 21. Function fitting analysis performed, with a calculated d (offset) = 7.7017(44381)820234, a = 1.203(99646), b = 0.115(22088), c = 4.173(33395) from fitting to pH = d + log_{10}(a* (I_{460}/I_{405})–b)/(c-(I_{460}/I_{405})).

To elucidate selectivity of the tripodal thiourea, and the effect of varying ionophore loading on the efficacy of cross-bilayer pH dissipation, the transporter stock solution (10 µL total) was varied as follows. From this, the fractional fluorescence intensity (I_{460}/I_{405}) (ESI 19) was converted to absolute pH using ESI 20 & 21.

1. 10 µL DMSO
2. 10 µL 1 mol% CCCP (concentration 0.0625 mM in DMSO, final concentration of 0.3125 µM)
3. 10 µL 0.1 mol% T (concentration 0.00625 mM in DMSO, final concentration of 0.03125 µM)
4. 10 µL 1 mol% T (concentration 0.0625 mM in DMSO, final concentration of 0.3125 µM)

5. 10 μL comprised of 1 mol% T AND 1 mol% CCCP (5 μL of concentration 0.125 mM T in DMSO AND 5 μL of concentration 0.125 mM CCCP, final concentration of 0.3125 μM of each)
6. 10 μL comprised of 0.1 mol% T AND 0.1 mol% CCCP (5 μL of concentration 0.0125 mM T in DMSO AND 5 μL of concentration 0.0125 mM CCCP, final concentration of 0.03125 μM respectively)
7. 10 μL comprised of 1 mol% T AND 1 mol% CCCP (5 μL of concentration 0.125 mM T in DMSO AND 5 μL of concentration 0.125 mM CCCP to give a final concentration of 0.3125 μM of each) but instead using 0.5 M H_2SO_4 to lower pH rather than 1 M HCl (ion selectivity).
8. 10 μL comprised of 1 mol% T AND 1 mol% CCCP (5 μL of concentration 0.125 mM T in DMSO AND 5 μL of concentration 0.125 mM CCCP to give a final concentration of 0.3125 μM of each) but instead using 1 M citric acid to lower pH rather than 1 M HCl (ion selectivity).

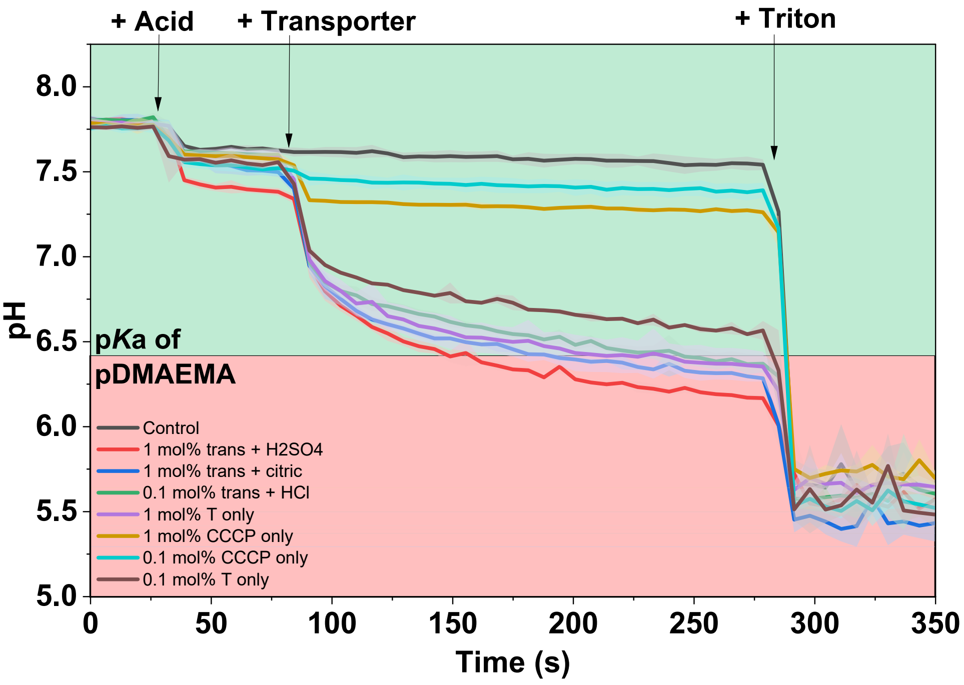

ESI 22. HPTS fluorescence assay data showing the quantified decrease in pH upon the incorporation of both ionophores (trans) or either cationophore (CCCP) or anionophore (T) only followed by external acidification in specific ion presence (*e.g.,* HCl, H_2SO_4, citric acid). Unless otherwise mentioned, HCl is pulsed in at 40 s. Importantly, DMSO addition (control test) shows no internal pH decrease, consistent with the high energetic barrier to ion passage in the absence of ion transporters. The pH dissipation for 1 mol% CCCP + tripodal thiourea in the presence of HCl is plotted in Figure 2 of the main paper (for comparison).

Synthesis of Cholesterol Modified POPC Vesicles for Lipid Bilayer and Polymer (Dual) Wrapped Gd-MSNs

1 mL of 5 mM K_2SO_4, pH 8.0 was added to a round bottom flask containing a film of dried lipids (266 μL 25 mg mL^{-1} POPC) with compositions of varying mol% cholesterol/POPC (typically 45 mol% cholesterol 55 mol% POPC). The mixture was then vortexed with 1 mL 5 mM K_2SO_4 pH 8.0 (+ KOH to basify) and subjected to 5 freeze-thaw cycles. The suspension was extruded 19 times through a 200 nm pore size polycarbonate membrane at room temperature, to afford a 1 mL suspension of cholesterol doped large unilamellar vesicles (LUVs).

Synthesis of bilayer wrapped pDMAEMA-Gd-MSNs (p-Gd-MSNs)

A 2.5 mg mL^{-1} solution of p-Gd-MSNs was sonicated in 1 mL of 5 mM K_2SO_4, pH 8.0. This solution was combined with 1 mL of the cholesterol doped LUV suspension. The mixture was then subjected to multiple short sonication and vortexing cycles. Excess vesicles (no encapsulated NP species) were removed through purification by centrifugation (10,300 x g for 20 minutes). The supernatant (excess LUVs) was discarded and the pellet (LB-p-Gd-MSNs) was redispersed in 1 mL 5 mM K_2SO_4 pH 8.0. This process was repeated a second time for further purification. The final solution was split into four portions, two at pH 8.0, and two at pH 5.0 (pH lowered using 0.1 N HCl solution). To one of the pH 8.0 and pH 5.0 portions each, 10 μL 1.25 mM tripodal thiourea in DMSO and 10 μL 1.25 mM CCCP (carbonyl cyanide m-chlorophenyl hydrazone) in DMSO were added (1 mol% each), and the other two portions were left unaltered (not mobile ion carrier modified). Finally, the bilayer-encapsulated polymer wrapped MSNs were left at 4 °C for relaxivity determination at 25 °C.

Consideration of TGA for vesicle-encapsulated pDMAEMA-Gd-MSNs (LB-p-Gd-MSNs)

TGA was used as a tool to confirm complete membrane wrapping of the LB-p-Gd-MSNs, by comparing the experimental mass lost with the theoretical mass lost. A calculated ~70,000 1-palmitoyl-2-oleoyl-glycero-3-phosphocholine (POPC) molecules form each liposomal envelope within this work. This equates to a mass of 8.83 x 10^{-17} g mol^{-1}. The total mass of the Gd-MSN, pDMAEMA coat and bilayer combined is 7.981 x 10^{-16} g mol^{-1}. As a fraction of the total mass, the POPC bilayer therefore accounts for a calculated 11.1% of the total mass of the dual layer encapsulated Gd-MSN. Accounting for the fact that the Gd-MSN loses 12% mass upon thermal ramping, the liposomal bilayer therefore accounts experimentally for 10.1% of the total mass, which is in line with the experimentally achieved value herein of 11.1% by TGA analysis.

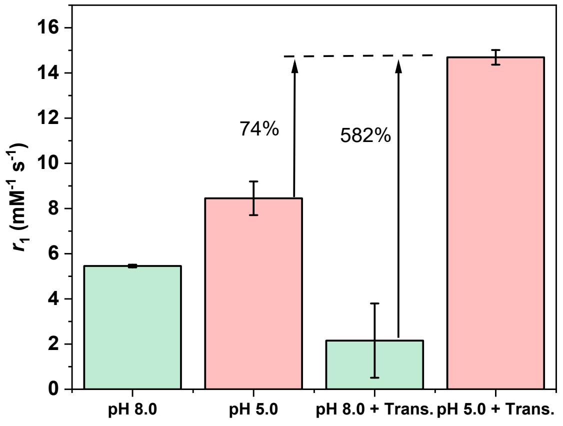

ESI 23. Longitudinal relaxivity analysis (measurements at 298 K, 1.41 T) of 30 mol% cholesterol 70 mol% POPC LB-p-Gd-MSNs, in the absence and presence of both CCCP and a tripodal thiourea motif, measured at pH 8.0 and 5.0 respectively. At pH 8.0, in the presence of both carriers, a further silencing in MRI contrast was observed, compared with no ionophore integration. The presence of ionophores enables the intravesicular space to further re-basify (full pH dissipation between internal and bulk solution) when re-suspended in pH 8.0 after purification by centrifugation, further inhibiting diffusive water access and supporting the presence of a more tightly wrapped polymer at pH > pK_a.[6] A more in-depth theoretical analysis utilising Solomon-Bloembergen-Morgan (SBM) theory to support the hypothesised origin of the relaxivity switching observed in this work is discussed in this ESI.

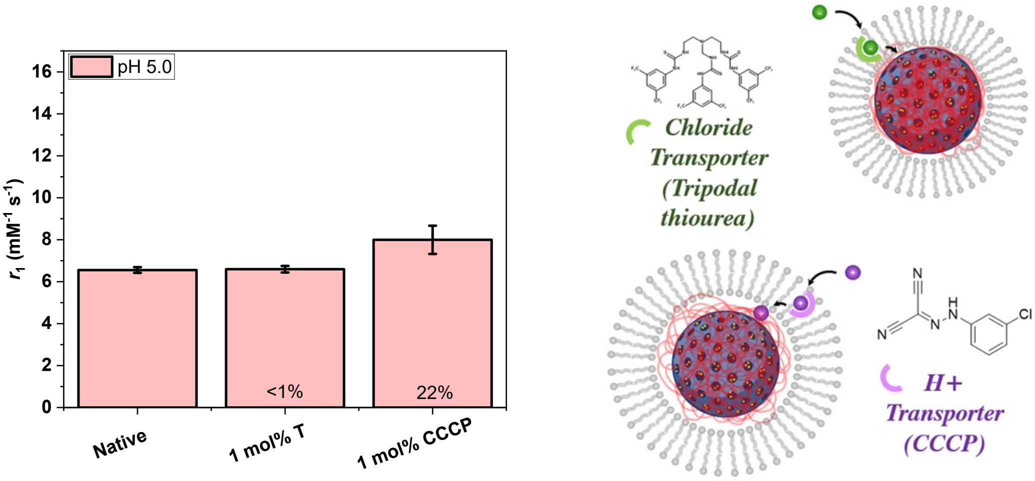

ESI 24. Longitudinal relaxivity analysis (measurements at 1.41 T, 298 K) of 45 mol% cholesterol 55 mol% POPC LB-p-Gd-MSNs, in the presence of 1 mol% CCCP and 1 mol% tripodal thiourea motif (T) individually, measured at pH 5.0. This displays that both ion carriers are necessary to generate the greatest switch in MRI contrast, due to the need for electroneutrality of transmembrane transport.[7]

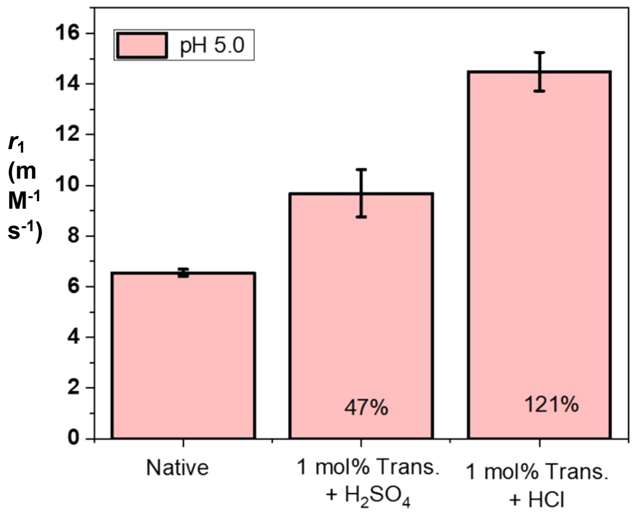

ESI 25. Longitudinal relaxivity analysis (298 K, 1.41 T) of 45 mol% cholesterol LB-p-Gd-MSNs, in the presence of 1 mol% CCCP and 1 mol% tripodal thiourea motif individually, measured at pH 5.0, varying specific anion presence from Cl^- to solely SO_4^{2-}.

Synthesis of Cholesterol Modified POPC Vesicles for Lipid Bilayer Wrapped Gd-MSNs

1 mL of 50 mM K_2SO_4, pH 8.0 (+ KOH to basify) was added to a round bottom flask containing a film of dried lipids (45 mol% cholesterol, 55 mol% POPC; 266 µL 25 mg mL^{-1} POPC). The mixture was then vortexed with the added 1 mL solution and subjected to 5 freeze-thaw cycles. The suspension was extruded 19 times through a 200 nm pore size polycarbonate membrane at room temperature, to afford a 1 mL suspension of cholesterol doped large unilamellar vesicles (LUVs).

Synthesis of bilayer wrapped Gd-MSNs (LB-Gd-MSNs)

A 2.5 mg mL^{-1} solution of Gd-MSNs was sonicated in 1 mL of 50 mM K_2SO_4, pH 8.0. This solution was combined with 1 mL of the cholesterol doped LUV suspension. The mixture was then subjected to multiple short sonication and vortexing cycles. Excess vesicles were removed through purification by centrifugation (10,300 x g for 20 minutes). The supernatant (excess LUVs) was discarded and the pellet (LB- Gd-MSNs) was redispersed in 2 mL 50 mM K_2SO_4 pH 8.0. This process was repeated a second time for further purification. The product was suspended in 1.6 mL of 50 mM K_2SO_4 pH 8.0, which was split into four portions, two at pH 8.0, and two at pH 5.0 (pH lowered using 0.01 N HCl solution). To one of the pH 8.0 and pH 5.0 portions each, 10 µL 1.25 mM tripodal thiourea in DMSO and 10 µL 1.25 mM CCCP (carbonyl cyanide m-chlorophenyl hydrazone) in DMSO were added (1 mol% each), and the other two portions were left unaltered (not mobile ion carrier modified). Finally, the bilayer-encapsulated Gd-MSNs were left at 4 °C for relaxivity determination at 25 °C (ESI 26).

Solomon-Bloembergen-Morgan (SBM) Theory Discussion/Consideration

The relaxivity shifts observed in this work are attributed to the modulation of the water pool available to the paramagnetic centres.[8] Gd-MSNs, when in the coated state, have significantly limited water pools in the vicinity of the individual Gd-DOTA moieties confined within the pores. Immediately after the magnetic pulse is applied, the Gd(III) centres have a 100% chance to encounter a perturbed spin, however, this chance continually decreases until the next pulse is applied. When the pool of water is very large (*i.e.,* a particle in the uncoated state) compared to the amount of contrast agent, the chance would remain close to 100%, thereby not decreasing the r_1 value. The approximate effects of a decreased water pool are modelled herein using SBM theory by modulating the effective inner-sphere residence time (τ_M) parameter within the calculation to achieve an appropriate fit.[9-11] The value of τ_M for a native Gd-MSN is reported to be 480 ns, whereas the *effective* τ_M calculated for the pH 8.0 native, pH 5.0 native, pH 8.0 + transporters, and pH 5.0 + transporters are 2100 ns, 2120 ns, 5700 ns, and 1050 ns, respectively. These values exhibit the significant effects of a small water pool on the ability of a Gd(III) centre to associate with a perturbed spin. ESI 27 shows the residence lifetimes dependent relaxivity of a LB-p-Gd-MSN for pH > pK_a or pH < pK_a, with and without ionophore integration. The other relevant values held constant for these approximations are as follows: $F^2 = 0.9$, $\tau_{RL} = 2.4 \times 10^{-9}$, $\tau_{RG} = 1 \times 10^{-4}$, $r_{Gd\text{-}H\,(IS)} = 3.1 \times 10^{-10}$, $B = 1.4$, $\tau_V = 1.5 \times 10^{-11}$, $q_{IS} = 1.1$, $q_{SS} = 4.3$, $\tau_{M(SS)} = 2.74 \times 10^{-11}$, $r_{Gd\text{-}H\,(SS)} = 3.6 \times 10^{-10}$, $r_{Gd\text{-}H\,(OS)} = 5.0 \times 10^{-10}$, $D = 3.1 \times 10^{-10}$.

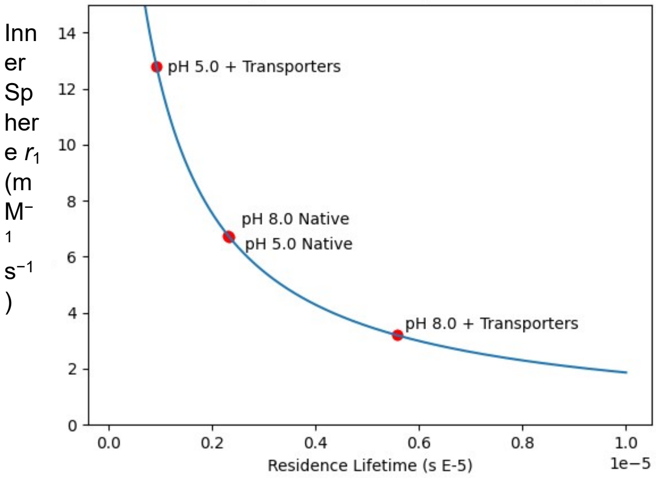

ESI 26. The relaxivity values observed for the particle systems fitted to the curve of inner sphere relaxivity vs. the residence lifetime of the directly coordinated water molecule to the Gd(III) centre. As referenced, the true value of residence lifetime is not likely to change, but when considered as the time taken, on average over the acquisition time, for the Gd(III) centre to associate with a perturbed water molecule, the changes to the residence lifetime mirror the relaxivity shifts observed.

Sample	H_2O (A)	LB-p-Gd-MSNs pH 8.0 no transporters (B)	LB-p-Gd-MSNs pH 5.0 + 1 mol% Tripodal Thiourea + 1 mol% CCCP (C)
T_1 / ms	3020 ± 15	2603 ± 19	1778 ± 9

(A) **(B)** **(C)**

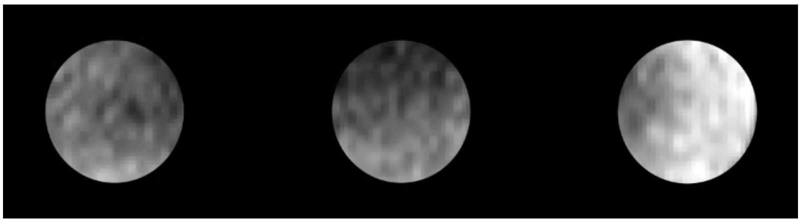

ESI 27. Raw MRI phantom images (T_1 maps) of (A) pH 7.0 aqueous solution; (B) LB-p-Gd-MSNs pH 8.0; and (C) LB-p-Gd-MSNs pH 5.0 + ionophores; taken at 4.7 T (298 K), showing the expected large decrease in T_1 time upon acidification from pH 8.0 to 5.0 and integration of both ionophores (3020 ± 15, 2603 ± 19, and 1178 ± 29 ms, respectively).

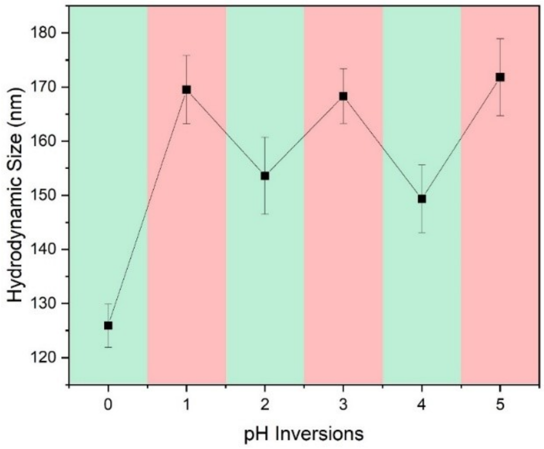

ESI 28. DLS data for the LB-p-Gd-MSNs upon 3 pH switching cycles (8.0, then 5.0 (x3)). The dual wrapped particles demonstrate a reversible swelling/deswelling upon alternating between acidic and basic conditions, due to the influx/efflux of H^+Cl^-. The error bars are reported as ± 1 s.d. either side of the mean value from three repeat measurements.

References:

1. D. Yuan, C. M. Ellis, F. E. Mózes and J. J. Davis, *Chem. Commun.*, 2023, 59, 6008–6011.
2. J. J. Davis, W. Y. Huang and G. L. Davies, *J. Mater. Chem.*, 2012, 22, 22848–22850.
3. M. R. Bhambhani, P. A. Cutting, K. S. W. Sing and D. H. Turk, *J. Colloid Interface Sci.*, 1972, 38, 109–117.
4. E. Grählert and M. J. Langton, *Angew. Chem. Int. Ed.*, 2025, 64, e202421580.
5. A. M. Gilchrist, P. Wang, I. Carreira-Barral, D. Alonso-Carrillo, X. Wu, R. Quesada and P. A. Gale, *Supramol. Chem.*, 2021, 33, 325–344.
6. D. E. S. Santos, D. Li, M. Ramstedt, J. E. Gautrot and T. A. Soares, *Langmuir*, 2019, 35, 5037–5049.
7. T. I. Rokitskaya, T. M. Ilyasova, I. I. Severina, Y. N. Antonenko and V. P. Skulachev, *Eur. Biophys. J.*, 2013, 42, 477–485.
8. A. M. Duncan, C. M. Ellis, H. Levingston, A. Kerckhoffs, F. E. Mózes, M. J. Langton and J. J. Davis, *Chem. Sci.*, 2024, 15, 13937-13941.
9. P. Caravan, C. T. Farrar, L. Frullano and R. Uppal, *Contrast Media Mol. Imaging*, 2009, 4, 89–100.
10. L. Tei, G. Gugliotta, Z. Baranyai and M. Botta, *Dalton Trans.*, 2009, 9712–9714.
11. L. M. De León-Rodríguez, A. F. Martins, M. C. Pinho, N. M. Rofsky and A. D. Sherry, *J. Magn. Reson. Imaging*, 2015, 42, 545–565.
